# LncRNA CCAT2, involving miR-34a/TGF-β1/Smad4 signaling, regulate hepatic stellate cells proliferation

**DOI:** 10.1038/s41598-022-25738-6

**Published:** 2022-12-08

**Authors:** Haibing Gao, Xiangmei Wang, Huaxi Ma, Shenglong Lin, Dongqing Zhang, Wenjun Wu, Ziyuan Liao, Mengyun Chen, Hanhui Ye, Qin Li, Minghua Lin, Dongliang Li

**Affiliations:** 1 Mengchao Hepatobiliary Hospital of Fujian Medical University, Fuzhou, 350025 Fujian Province China; 2 Fuzhong Clinical Medical College of Fujian Medical University, Fuzhou, 362002 Fujian Province China; 3 900th Hospital of Joint Logistics Support Forces of the Chinese PLA, Fuzhou, 350000 Fujian Province China

**Keywords:** Cell biology, Molecular biology

## Abstract

miR-34a targeting on Smad4 plays important role in TGF-β1 pathway which is a dominant factor for balancing collagen production and degradation in hepatic stellate cells. TGF-β1/Smad4 regulated collagen deposition is a hallmark of hepatic fibrosis. The potential regulation on miR-34a by LncRNAs in hepatic stellate cells (HSCs) is still reserved to be revealed. In current study, it was hypothesized that a miR-34a interactor, lncRNA CCAT2 may regulate TGF-β1 pathway in liver fibrotic remodeling. The interaction between CCAT2 and miR-34a-5p was checked by dual luciferase assay. the effects of CCAT2 and miR-34a-5p on cell proliferation and apoptosis were verified by MTT assay, colony formation assay, and flow cytometry assay. Dual luciferase activity showed CCAT2 are targets of miR-34a-5p. Sh-CCAT2 transfection prohibit HSCs proliferation and induce HSCs apoptosis, also inhibited ECM protein synthesis in HSCs. Decreased miR-34a-5p enhanced HSCs proliferation, blocked HSCs apoptosis and promoted ECM protein production. miR-34a-5p inhibitor undo protective regulation of sh-CCAT2 in liver fibrosis. Furthermore, clinical investigation showed that CCAT2 and Smad4 expression level were significantly induced, while miR-34a-5p was significantly decreased in HBV related liver fibrosis serum. In conclusion, activated HSCs via TGF-β1/Smad4 signaling pathway was successfully alleviated by CCAT2 inhibition through miR-34a-5p elevation.

## Introduction

Multiple evidences proved that miR-34a plays important role in multiple physiology processes^1–5^.

It was proved that *transforming growth factor beta 1*(*TGF-β1*)/*Smad4* is target of miR-34a^6–8^. TGF-β1 is regarded as the crucial signaling pathway in liver fibrosis^9^. TGF-β1 is distributed in most human tissues and attenuates cell proliferation, differentiation, migration and apoptosis^10^. As Smad proteins are transcriptional factor of TGF-β1 expression, TGF-β1/Smad signaling in liver fibrosis has been broadly studied. Inhibition of SMAD3 decreased collagen I expression while Smad2 increased collagen I expression, and Smad4 is crucial in liver fibrosis by supporting SMAD3 activity^11^. TGF-β1 modulate liver fibrosis by activating Smad2 and Smad3 pathway, whereas Smad7 is known as an inhibitor of TGF-β1^12^. Although role of TGF-β1 in liver fibrosis has been carried out, role of its interactor miR-34a in liver fibrosis is still unclear. Studies have been discovered that miR-34a-5p was increased and play function in progress of the fibrotic disease. Feili et al., proved that miR-34-5p level was upregulated and enhanced HSCs activation^13^. Also, Ibusuki et al., concluded that miR-34a-5p was elevated by Human Neutrophil Peptide-1 (HNP-1) secretion and enhanced hepatocyte apoptosis which result in liver fibrosis^14^.

Liver is a largest internal solid organ of the human body, which plays pivotal in innate immunity against pathogens such as microorganisms, chemicals and antigens^15^. Continuous exposure to toxic substances or chronic liver damage over long period and related wound repairing process generate insufficient extracellular matrix (ECM) protein accumulation^16^. Fibroblast-specific protein 1(FSP1) is usually found in lung, kidney and liver, which goes through tissue fibrogenesis^17^. α-SMA is also known as a considerable marker of activation of HSCs and progression of fibrosis^18,19^. ECM, especially collagen type I production is a major feature of activation of HSCs and it is regulated by various stimuli and signaling pathways^20^.

Non-coding RNAs (miRNAs, lncRNAs) have been emerging as a therapeutic targets, because it is participated in almost all biological process in tissue specific manner^21,22^. LncRNAs are involved various mechanisms, including transcription regulation, translation, protein modification and RNA–protein or protein–protein formation, so that lncRNAs are able to control biological processes^23^. Several lncRNAs including HOTAIR, MEG3, H19, GAS5, lncRAN-COX-2, APTR and lnc-LFAR1, showed statistically different expression in liver fibrosis^24^. Since, Ling et al. first identified lncRNA colon cancer associated transcript 2 (CCAT2) which control tumorigenesis in colon cancer^25^, subsequent studies have been revealed CCAT2 involved in different types of cancer progression including gastric, lung, colorectal, breast and hepatocellular carcinoma^26–32^. In addition, previous studies exhibited that CCAT2 activates cancer progression and metastasis through the TGF-β1 signaling pathway^26,33^. However, the contribution of CCAT2 and TGF-β1 signaling pathway in liver fibrosis and its intrinsic mechanism are not yet investigated.

Thus, reviewing relative literatures, we hypothesized that lncRNA CCAT2 regulates TGF-β1/Smad signaling via sponging miR-34a-5p in hepatic fibrogenesis. Here, CCAT2, miR-34a-5p and Smad4 expression level in hepatitis B virus (HBV) related fibrosis were evaluated. Also, the mechanism of CCAT2, miR-34a-5p and TGF-β1/Smad signaling pathway on HSCs cell proliferation, cell cycle and collagen deposition related protein expression were revealed.

## Materials and methods

### Clinical specimens

The liver fibrosis patients with HBV and healthy population was recruited during January 2019 to December 2019 from Mengchao Hepatobiliary Hospital of Fujian Medical University. study has been approved by the ethics committee of Mengchao Hepatobiliary Hospital of Fujian Medical University. Total fifty with HBV and fifty for control were enrolled (aged 18–65 years, presence of HBV DNA < 10^3^ IU/mL, α-fetoprotein ≤ 20 ng/m). If they had co-infection with hepatitis C, D and E virus or other type of hepatitis, cirrhosis, HCC, or with severe heart, kidney and brain disease, patients were also excluded. All participants were provided informed verbal and written consent and signed on consent form.

### Cell culture and transfection

LX-2 cells were purchased (Merck Millipore; Billerica, MA) and were cultured in DMEM (plus 5% FBS) (Hyclone, Logan, Utah, USA) Cells were incubated 37 ℃ and 5% CO_2_. Cell transfection were proceed as following step: 1 Cell plateing: one day before the transfection operation, inoculate cells with an appropriate density in a six well plate; 2. Medium change: Before the transfection operation, the old medium in the six hole plate should be discarded and replaced with a fresh medium containing 2 ml serum but no antibiotics; 3. Transfection: prepare twice as many sterile 1.5 ml EP tubes according to the number of holes in the six hole plate. For each hole cell, add 125uL pure DMEM culture solution (without antibiotics and serum) in tube A and tube B respectively, add 2.5ug plasmid DNA in tube A, and gently blow and suck them with a pipette gun; Add a certain amount of Lipo6000 transfection reagent (5ul) into tube B, blow, suck and mix DMEM and Lipo6000 equally, incubate them at room temperature for five minutes, then suck the solution in tube A with plasmid DNA and add it into the mixed solution containing transfection reagent in tube B, blow, suck and mix the two tubes of solution with a pipette gun, and drop the mixed solution into a six hole plate after standing at room temperature for 20 min. Transfection ratio more than 70% were prepared for further experiments.

### Western blot

(1) Extraction of total protein (2) gum making and gel running; (4) Turn the membrane. With reference to the size of separation gel, cut out filter paper and NC membrane of the same size, place them in the order of black gel and white membrane, and place them in the membrane transfer tank. Then assemble the instrument, set the parameter to 200 mA, and turn the film for 45 min. (5) Closed. After the membrane conversion process, soak the protein side upwards in 5% skimmed milk, and seal it on a low-speed shaking table at room temperature for 1 h. (6) Hybridization. Use diluted 1 × TBST rinses the closed NC membrane. Then, dilute the first antibody (sigma) with 5% skimmed milk, cover the first antibody with the NC membrane facing down, and incubate it at 4 ℃ overnight. Then use 1 again the next day × TBST rinses NC membrane for three times. Then, the second antibody (sigma) was incubated in the incubator according to the same operation, and incubated in the shaking table at room temperature for 1 h. (7) Chemiluminescence. Take out the NC membrane after incubation, and use 1 × TBST rinsing for three times. Take the same amount of developer A and B, mix them evenly, drop them on the NC film, incubate them in dark for 5 min, develop and image in the gel imager, and take photos.

### Real time qRT-PCR

Total RNA was purified by NucleoZol reagent (MACHEREY–NAGEL, Germany). 1μL of extracted total RNA was converted to cDNA by Prime Script TM RT Master Mix (TaKaRa Bio Technology, Dalian, China). Real-time qPCR was carried out on ABI 7500 (Applied Biosystems, Inc., Foster City, CA) according to the manufacturer’s manual. Primers were purchased from Ribo (Guangzhou, China). Following primers were used: (1) CCAT2, forward: 5′-TGGACTGGAAGTCAAGAGCC-3′, reverse: 5′-CCCAGATGCAGAGAACGAGG-3′. (2) Smad4, forward: 5′-CCAGCTCTGTTAGCCCCATC-3′, reverse : 5′-TACTGGCAGGCTGACTTGTG-3′. (3) miR-34a-5p, forward : 5′-CGCGTGGCAGTGTCTTAGCT-3′, reverse : 5′-AGTGCAGGGTCCGAGGTATT-3′, RT Primer : 5′-GTCGTATCCAGTGCAGGGTCCGAG GTATTCGCAC TGGATACGACACAACC-3′. (4) GAPDH, forward : 5′-GTCATCCCTGAGCTGAACGG-3′, reverse : 5′-CCACCTGGTGCTCAGTGTAG-3′. 2^-ΔΔCt^ method was used to quantitative analysis of the data compared to control. The internal control gene was GAPDH.

### MTT assay

In the first step, 96-well plates was used to grow cells at 5 × 10^4^ cells per well. Incubated at a 37 ℃ incubator and added 20 μl of MTT. The concentration of MTT was 5 mg/ml. After 0, 24, 48 and 72 h culturing, medium was changed to 150 μl DMSO, shook well for ten minutes. Absorbances were measured at OD490. All analyses were performed in triplicates.

### Flow cytometry cell analysis

After 48 h of infection, cells were centrifuged and collected and fixed with 70% alcohol at 4 ℃ for 24 h. Centrifuge the cells again, and wash the cells with 1 ml of PBS, 500 μl of 1 × binding buffer and 50 μl of PI (50:1). Incubated in a dark room for 30 min at room temperature. Then flow cytometry detection were carried out by ModFit LT software. FL2-w and FL2-A were used to display and remove conjoined cells.

### Colony formation

Transfected 800 cells were maintained in medium containing 10% of FBS. Shake the transfected cells and place them in the incubator for culturing. Change the medium every 3 days and observe the cell status and colony size for two weeks. Colonies were fixed with 4% paraformaldehyde in a refrigerator at 4 ℃ for 60 min, and stained with crystal violet. Single colony with greater than 50 cells were counted.

### Dual luciferase reporter

Luciferase reporter plasmid was constructed and transfected cell by using Lipofectamine™ 2000 for 48 h. The signal was checked by using GLO-MAX 20/20 (Promega, Madison, USA).

### Statistical analysis

Data were displayed as the mean ± standard deviations. SPSS for Windows (v.13.0) was used for all analysis. The differences between groups were test by t-test. The statistically significant is judged by *p* < 0.05.

## Results

### CCAT2 promote cell proliferation and collagen precipitation, inhibit apoptosis

**Figure 1:**
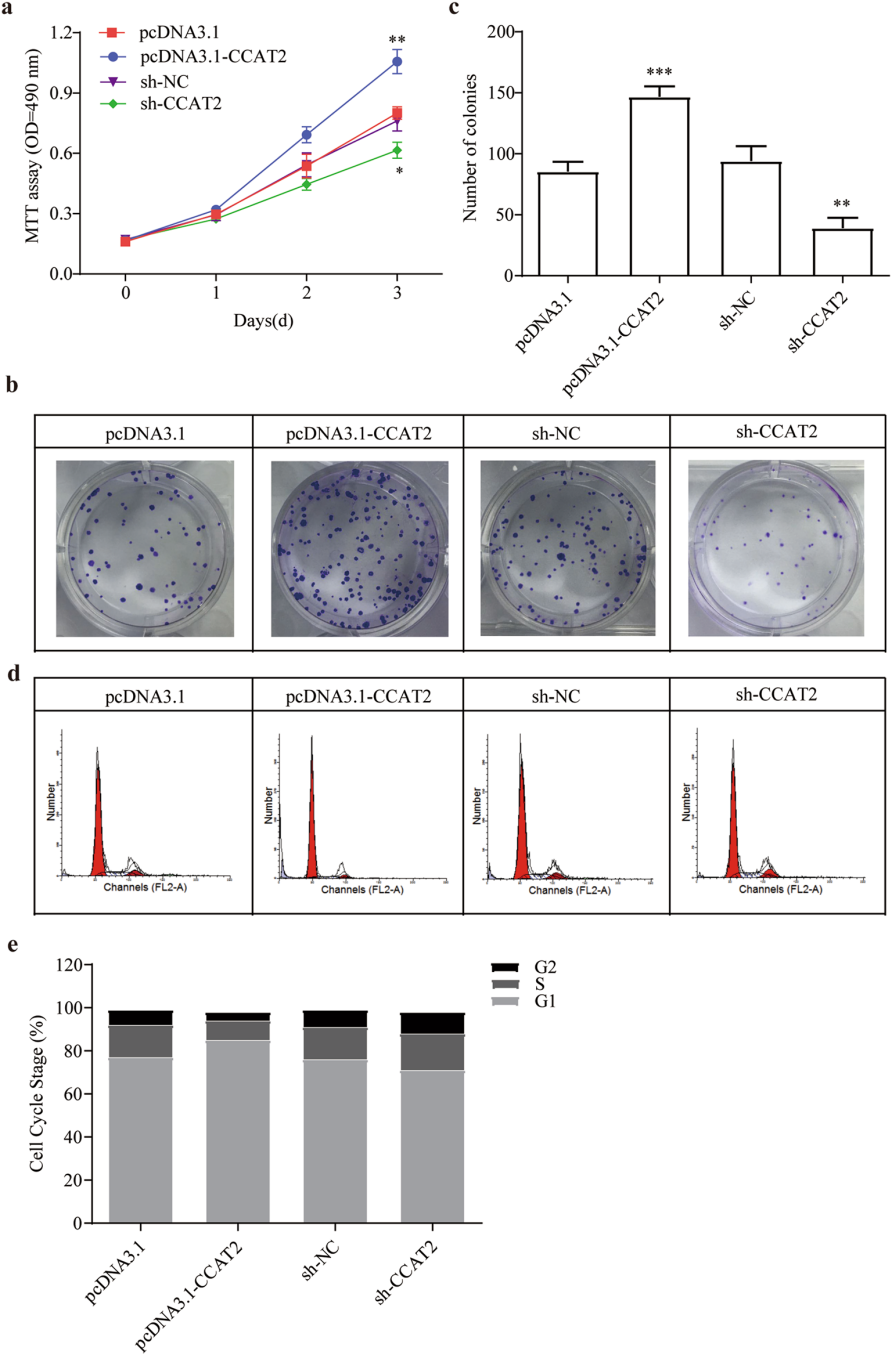
CCAT2 effects on lx-2 cell proliferation, collagen precipitation and apoptosis. (**a**) Comparison of cell viability of lx-2 cells transfected with sh-CCAT2 or pcDNA3.1-CCAT2 was determined by MTT assay. (**b**) and (**c**) Colony formation assay and the quantification. (**d**) and (**e**) Cell-cycle distribution by flow cytometry, and statistical analysis of distribution of lx-2cell cycle stages.

Results revealed HSCs which transfected with overexpressed CCAT2 plasmid remarkably increased cell proliferation (Fig. 1a, *p* < 0.01). Similarly, knockdown of CCAT2 significantly inhibited growth of HSCs cells (Fig. 1a, *p* < 0.05). Similar results were demonstrated by colony formation assay (Fig. 1b, c). Subsequently, cell-cycle assay was demonstrated to verify whether CCAT2 modulates HSCs cell cycle. Flow cytometry results are shown in Fig. 1d, e. Overexpressed CCAT2 showed increased G1 phase and shortened S and G2 phase. Silencing of CCAT2 in the HSCs cells induced decreased distribution of G1 phase. Supplemental Fig. 1 showed the cell clycle and apoptosis marker protein changed according to above results. These findings suggested sh-CCAT2 transfection in HSCs cells induced cell cycle arrestment in the G1 stage, and promoted cell apoptosis.

**Figure 2:**
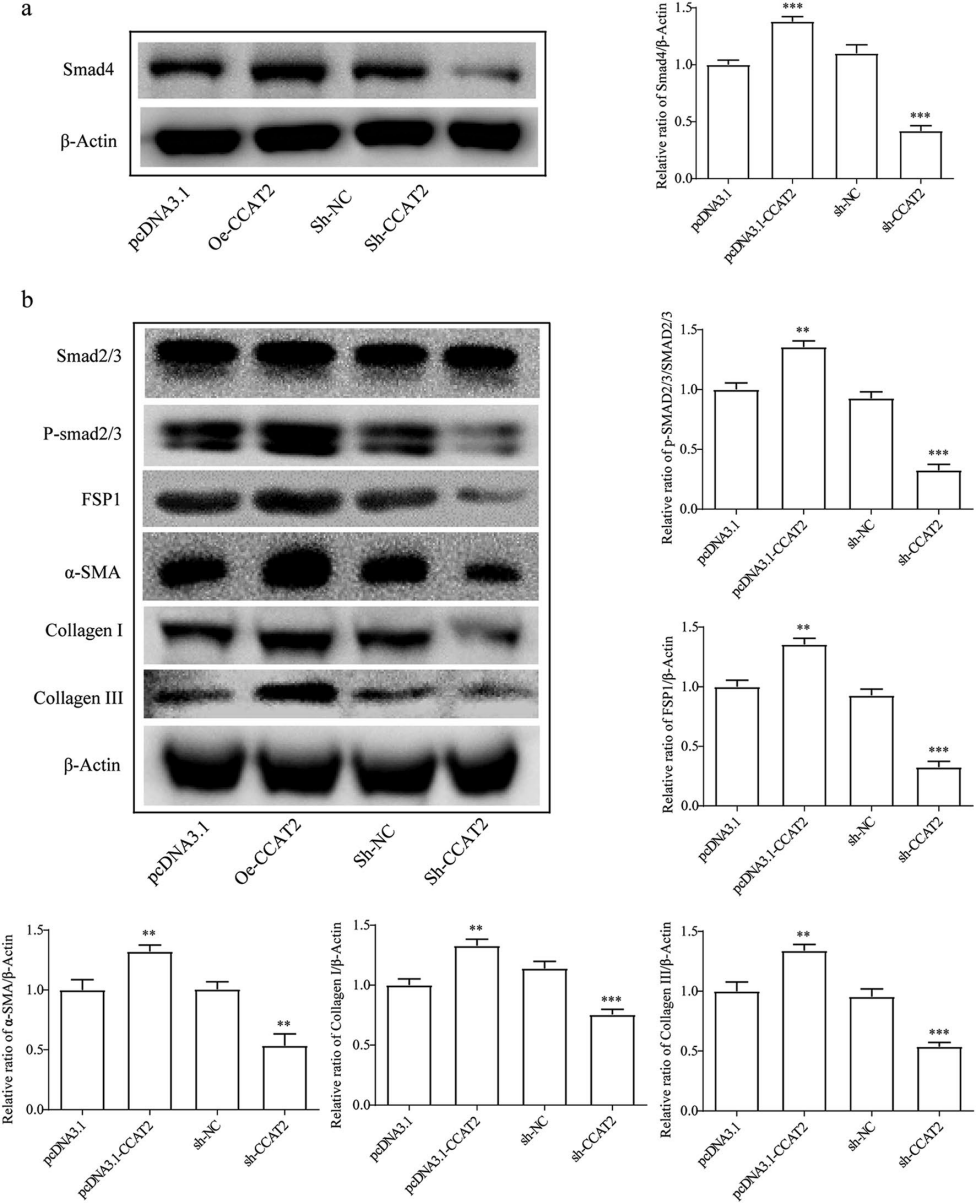
CCAT2 regulates EMT related proteins and Smad signaling in HSCs. β-actin was used as an internal control. Data are reported as means ± SD.

Then, to investigate the interrelationship between CCAT2 and Smad4 pathway, western blot was performed. Smad4 which is transfected with upregulated CCAT2 showed significantly increased protein expression level (Fig. 2a, *p* < 0.001). Also, fibrogenesis related ECM proteins and signaling pathway Smad2/3 (phosphorylated) were checked by western blot. As displayed in Fig. 2b, the western blot result showed phosphorylated Smad2/3, FSP1, collagens I and III and α-SMA protein level in the sh-CCAT2 group were significantly downregulated, indicating that knockdown of CCAT2 inhibited generation of ECM proteins production.

### CCAT2 is target of miR-34a-5p

**Figure 3:**
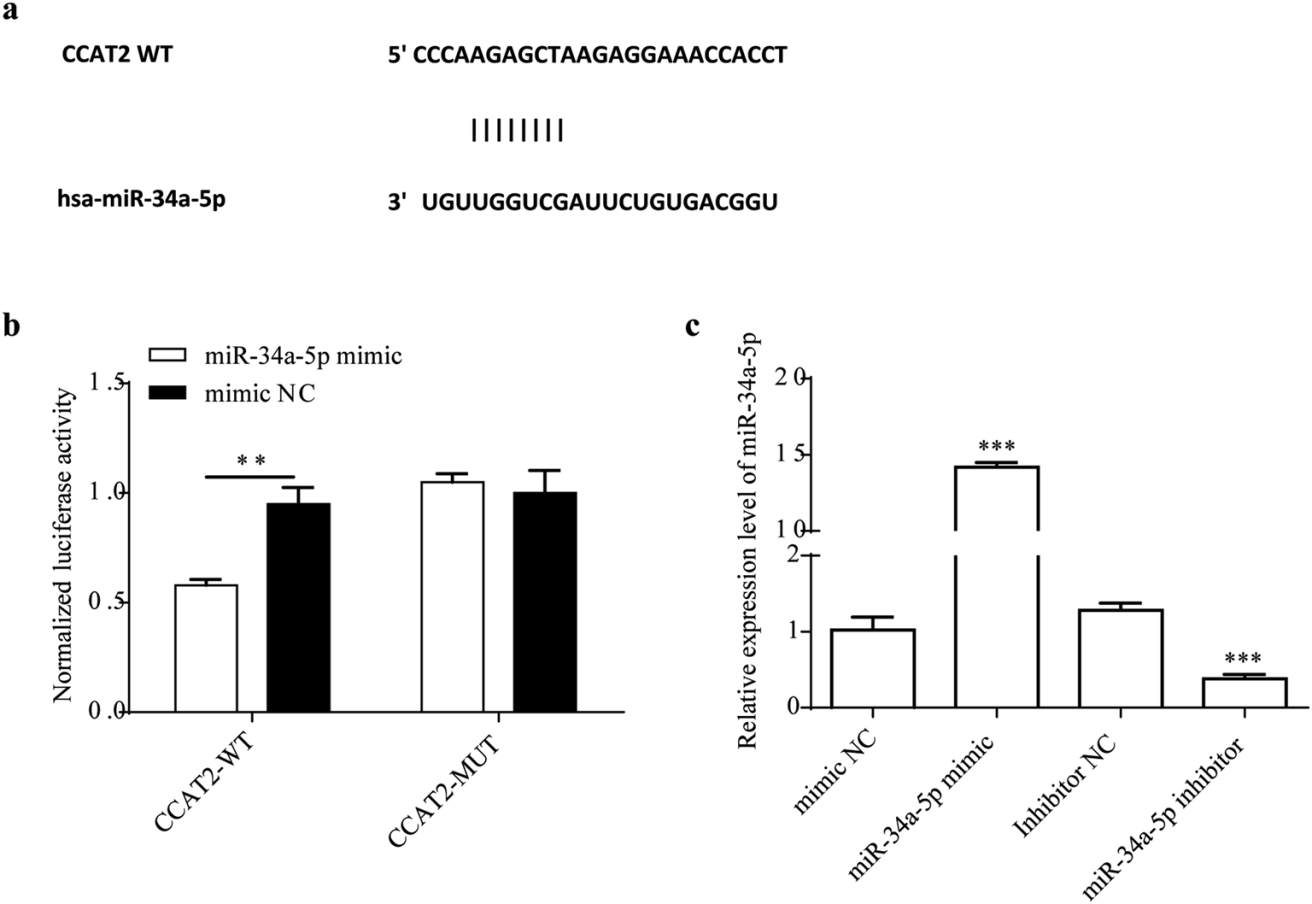
miR-34a-5p interact with 5′-UTRs of CCAT2. Dual luciferase assay of HSCs co-transfected with CCAT2 wild type or mutants and miR-34a-5p mimics or negative control.

Many studies have validated that lncRNA CCAT2 sponging miRNAs in different types of cancer^34–40^. In the 5′-UTR CCAT2, there is miR-34a-5p interaction sites (Fig. 3a). miR-34a-5p notable reduce the signal of vector containing CCAT2 wild type rather than CCAT2 mutant with mutation at predicted interaction sites (Fig. 3b, *p* < 0.01). It suggests that without this specific site, miR-34a-5p could not reduce the level of CCAT2 via interaction dependent silencing. In addition, RT-qPCR showed miR-34a-5p level in the miR-34a-5p inhibitor group was reduced, whereas the level of miR-34a-5p in the miRNA-34a-5p mimic group was up-regulated (Fig. 3c, *p* < 0.001, respectively). To sum up, all data indicated CCAT2 at 5′-UTR is a interaction site of miR-34a-5p and related negatively in HSCs.

### miR-34a-5p inhibits HSCs proliferation, collagen precipitation, induces apoptosis

**Figure 4:**
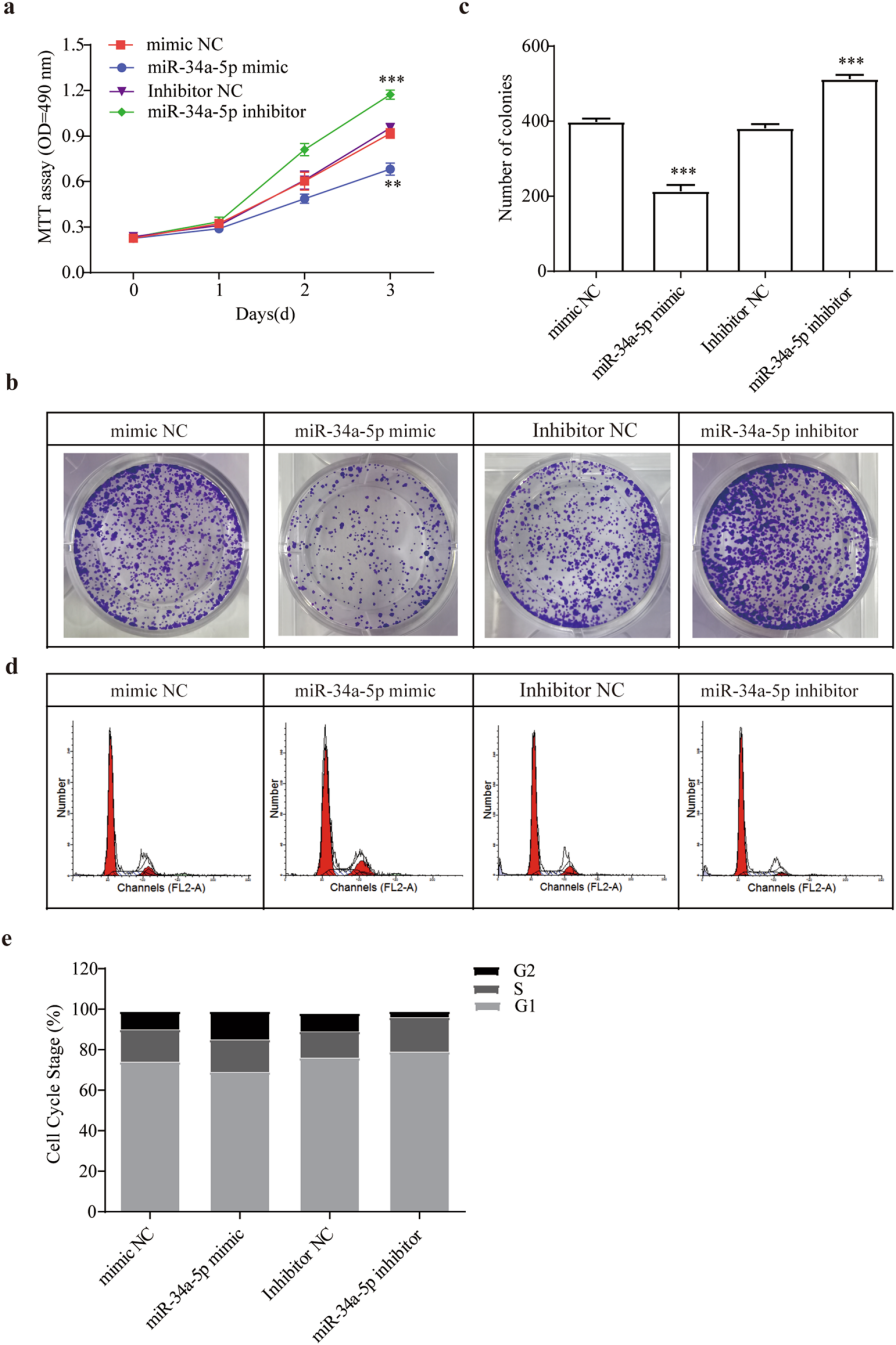
miR-34a-5p regulates lx-2 cell in proliferation, collagen precipitation and apoptosis. (**a**) Comparison of cell proliferation of HSCs transfected with miR-34a-5p mimic or inhibitor was determined by MTT assay. (**b**) Colony formation assay and (**c**) the quantification. (**d**) and (**e**) Cell-cycle distribution by flow cytometry, and statistical analysis of distribution of HSCs cell cycle stages.

Next, MTT assay and colony forming assay were done to discovery the cellular function of miR-34a-5p on HSCs. Test findings were revealed overexpression of miR-34a-5p remarkably prohibited HSCs growth (Fig. 4a, *p* < 0.01). Cell cycle analysis showed corresponding results with MTT analysis that increased of miR-34a-5p reduced HSCs growth, while inhibitor promoted HSCs growth (Fig. 4b–e, *p* < 0.001). Supplemental Fig. 2 showed the cell clycle and apoptosis marker protein changed according to above results.

**Figure 5:**
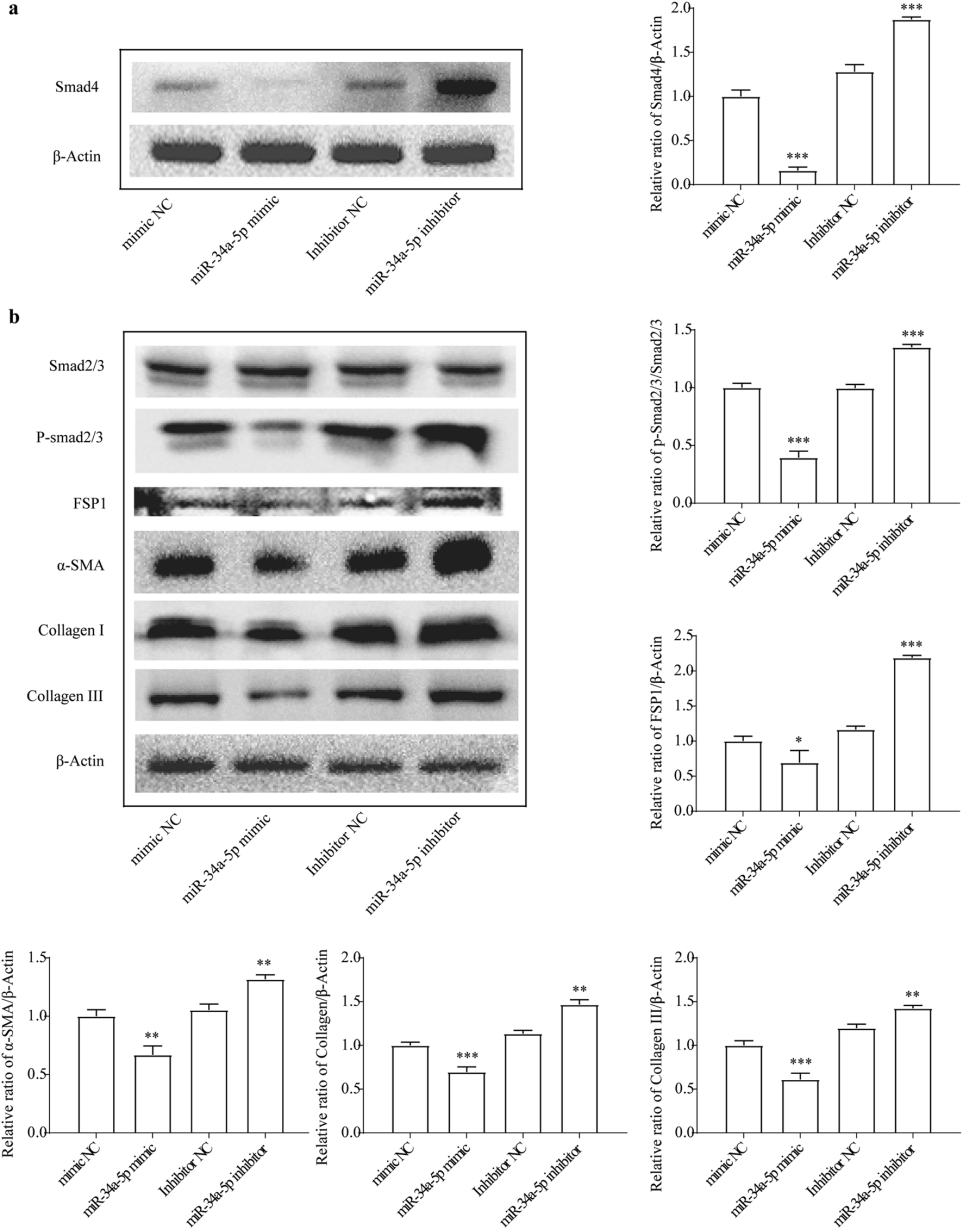
miR-34a-5p controls level of ECM related proteins and Smad signaling in HSCs. (**a**) Smad4 protein level changed after miR-34a-5p mimic and inhibitor treatment. (**b**) Smad signaling and ECM related proteins level changed after miR-34a-5p mimic and inhibitor treatment.*, *p* < 0.05, ** , *p* < 0.01,***, *p* < 0.005. β-actin was used as an internal control. Data are reported as means ± SD.

Western-blot results showed Smad4 and phosphorylation of Smad2/3 pathway were activated by miR-34a-5p inhibitor (Fig. 5). The ECM proteins such as collagens I, III, α-SMA and FSP1 were increased by suppressed miR-34a-5p (Fig. 5). Hence, we can conclude that miR-34a-5p inhibition cause enhancing the collagen production and ECM proteins, that is, silencing of miRNA-34a-5p can promote liver fibrosis.

### miRNA-34a-5p negatively modulated CCAT2

**Figure 6:**
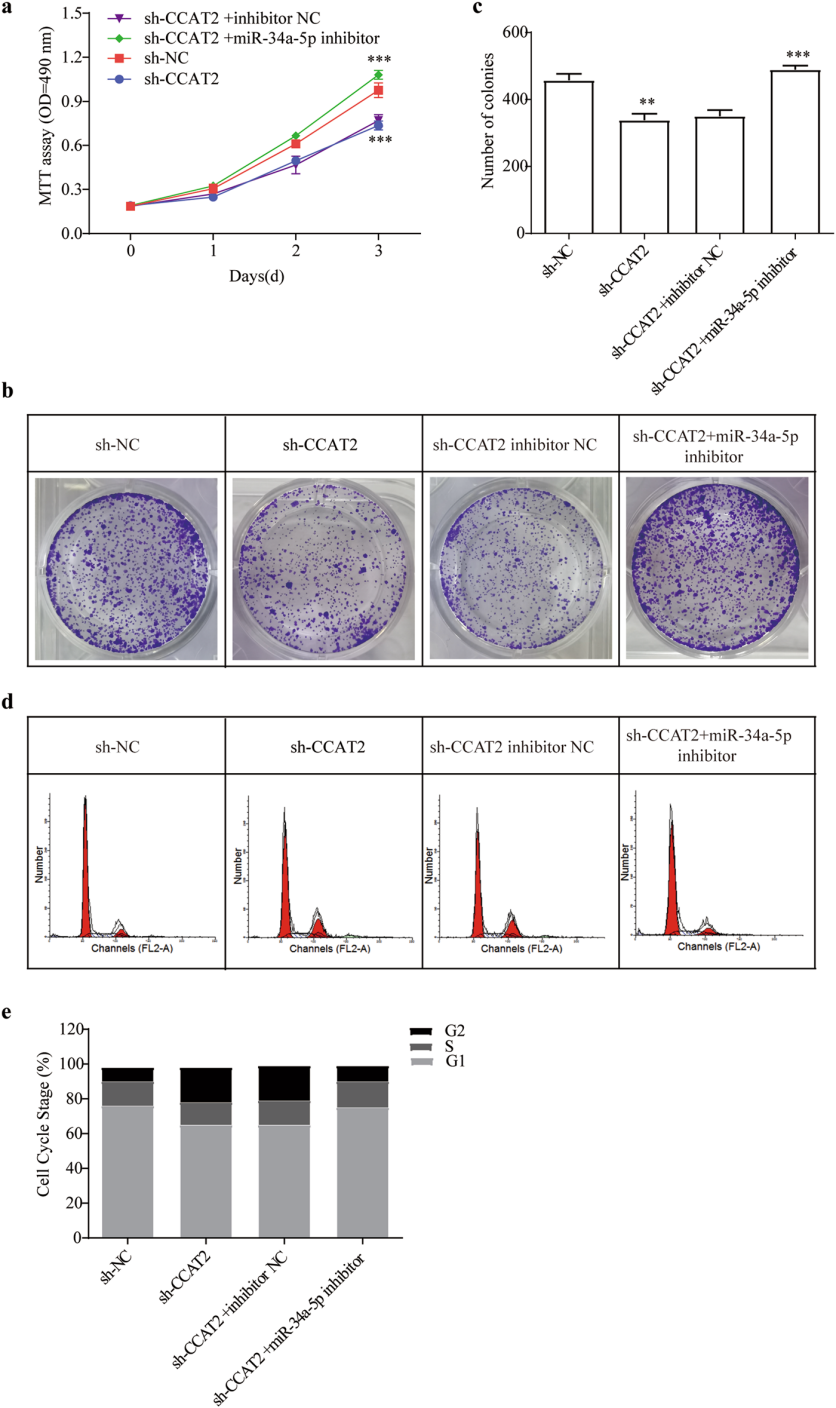
CCAT2 regulates lx-2 cells through miR-34a-5p. (**a**) Comparison of cell proliferation of HSCs co-transfected with sh-CCAT2 and miR-34a-5p mimic or inhibitor was determined by MTT assay. (**b**) Colony formation assay and (**c**) the quantification. (**d**) and (**e**) Cell-cycle distribution by flow cytometry, and statistical analysis of distribution of HSCs cell cycle stages.

**Figure 7:**
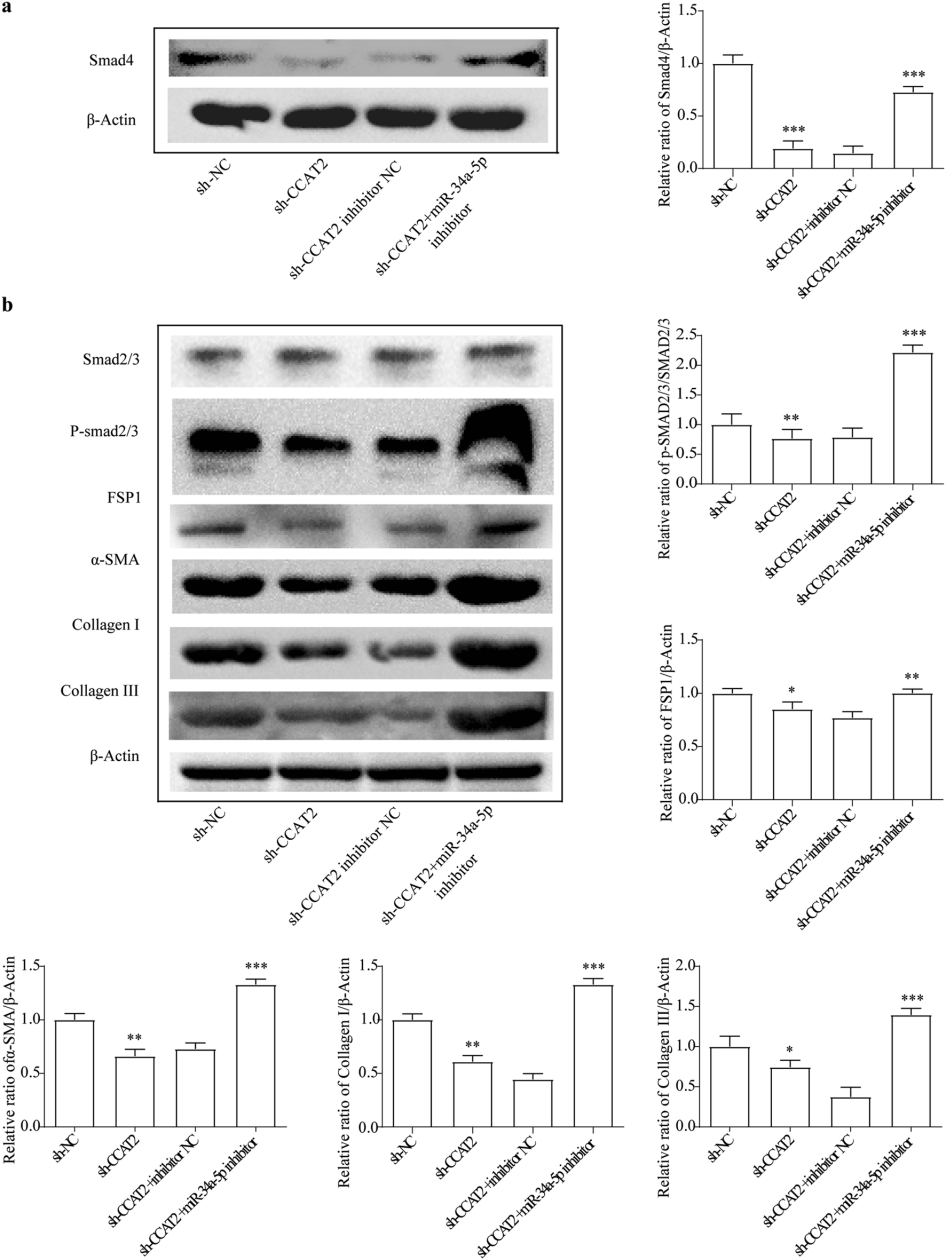
CCAT2 control miR34a-5p/Smad signaling axis. (**a**) Smad4 protein level were checked after miR-34a-5p mimic and inhibitor treatment. (**b**) Smad signaling and ECM related proteins level were checked after miR-34a-5p mimic and inhibitor treatment.*, *p* < 0.05, ** , *p* < 0.01,***, *p* < 0.005. β-actin was used as an internal control. Data are reported as means ± SD.

In order to role of CCAT2 interaction on miR-34a-5p in HSCs cells, series experiments were performed. Silence of the CCAT2 reduced HSCs proliferation, and combination of miR-34a-5p inhibitor and CCAT2 knockdown considerably increased cell viability (Fig. 6a, *p* < 0.001, respectively). Likewise, colony forming assay showed same effect on HSCs cell proliferation (Fig. 6b, c). The results of cell cycle experiments showed that the G1 phase of cells in the miR-34a-5p inhibitor and CCAT2 knockdown group was increased, the S phase and G2 phase were shortened, indicating that the cells had stronger activity (Fig. 6d, e). Supplemental Fig. 3 showed the cell clycle and apoptosis marker protein changed according to above results. Silenced CCAT2 along with suppressed miR-34a-5p plasmid successfully recovered expression levels of ECM proteins and Smad pathways (Fig. 7). MiR-34a-5p inhibitor reversed the protective sh-CCAT2 function.

### Downregulated of CCAT2 promotes TGF-β1 gene silence

**Figure 8:**
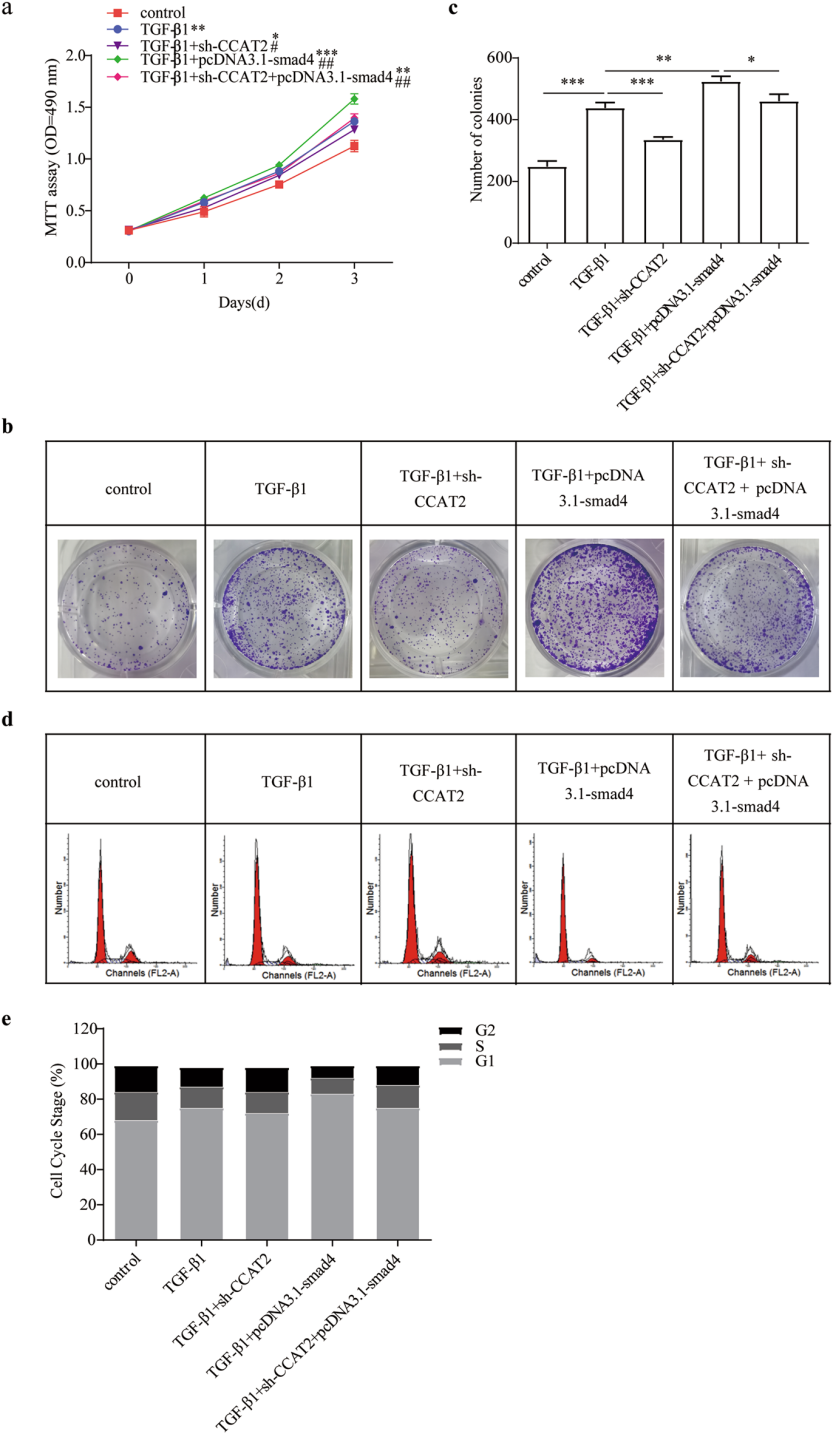
TGF-β1 and Smad4 are downstream signaling of CCAT2 in HSCs. (**a**) Comparison of cell proliferation of HSCs co-transfected with TGF-β1 and sh-CCAT2, pcDNA3.1-Smad4 was determined by MTT assay. (**b**) Colony formation assay and (**c**) the quantification. (**d**) Cell-cycle distribution by flow cytometry, and statistical analysis of distribution of HSCs cell cycle stages (**e**).

**Figure 9:**
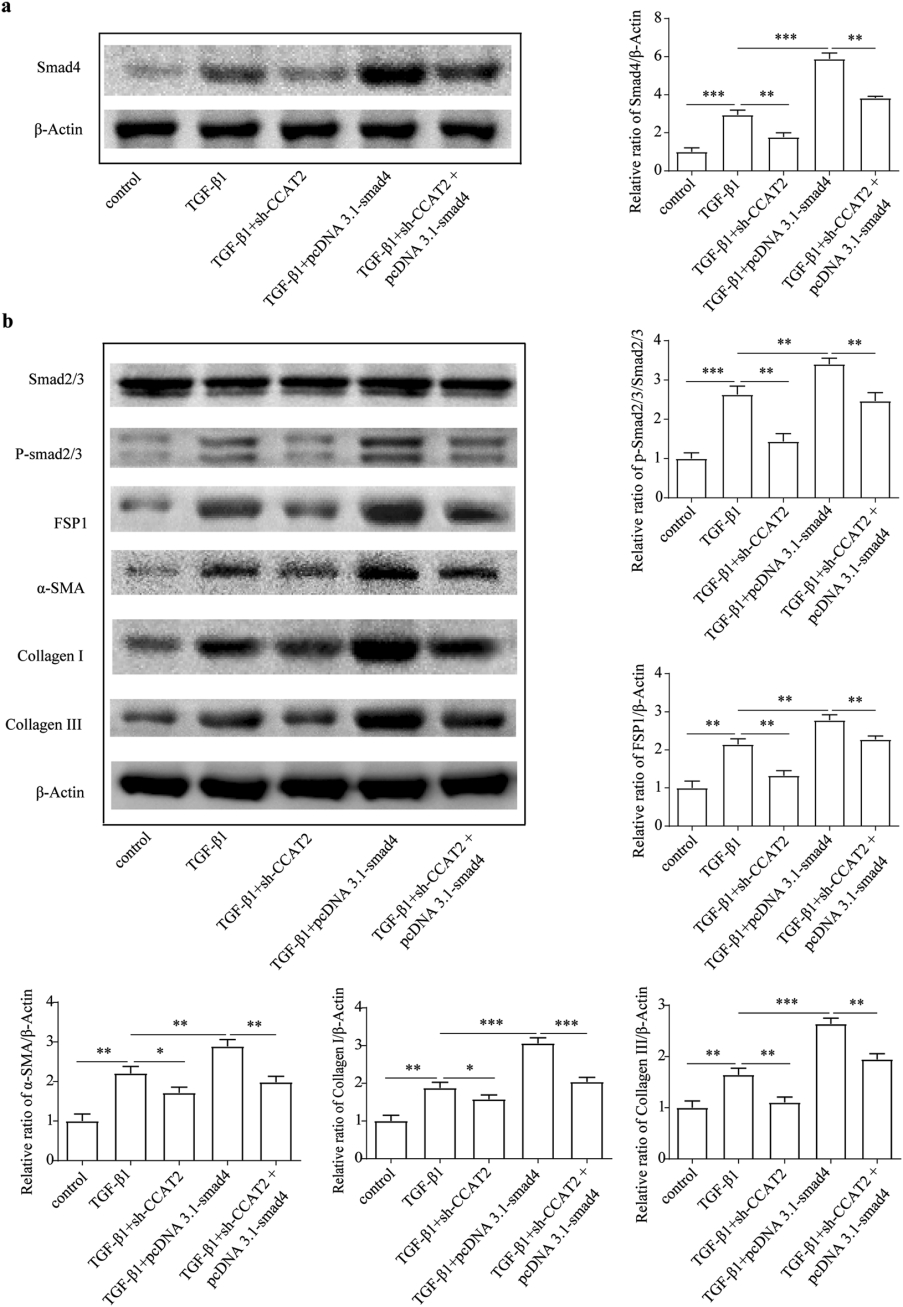
Related level of ECM related proteins and Smad signaling in HSCs regulated by CCAT2. (**a**) Smad4 protein level were determined after miR-34a-5p mimic and inhibitor treatment. (**b**) Smad signaling and ECM related proteins level were determined after miR-34a-5p mimic and inhibitor treatment.*, *p* < 0.05, ** , *p* < 0.01,***, *p* < 0.005. β-actin was used as an internal control. Data are reported as means ± SD.

It was well known that TGF-β1 is the crucial profibrogenic factor of liver fibrogenesis via activating HSCs, increasing collagen synthesis and inhibit collagen degradation^41^. HSCs cells were co-infected with TGF-β1 and Smad4 overexpression vector significantly increased activation of proliferation. However, addition of downregulated CCAT2 vector alleviated their fibrogenesis effect (Fig. 8a–c). Cell cycle experiments also further illustrate the above experimental results (Fig. 8d, e). Supplemental Fig. 4 showed the cell clycle and apoptosis marker protein changed according to above results. Sh-CCAT2 treatment successfully reduced ECM proteins and collagen synthesis (Fig. 9). Inhibited CCAT2 is negatively related to TGF-β1/Smad4 and TGF-β1/p-Smad2/3 pathway and suppressed HSCs activation.

### 3′-UTR of Smad4 interacted with miR-34a-5p

Supplemental Fig. 5 showed the interaction site in the 3′-UTR Smad4. Result showed that miR-34a-5p mimic luciferase activity was significantly decreased with Smad4 wild-type, which indicated that Smad4 3′-UTR is a miR-34a-5p target.

## Discussion

Liver fibrosis mostly caused by chronic viral hepatitis and liver injuries caused by alcohol abuse, leads to cirrhosis which shows poor prognosis and high mortality^42,43^. Liver cirrhosis is an outcome of constant injuries in liver for years, and it may affect major organ failure including kidney and brain^44^. Taking into consideration the burdens of cirrhosis, early detection and appropriate interventions can improve the disease prognosis before the fibrosis aggravated. In the clinical practice, anti-inflammatory or immunological medications including corticosteroids, phlebotomy for iron chelation and antiviral therapy for HCV and HBV are often prescribed^45–47^. In clinical research, molecular biomarkers and anti-fibrogenic target therapy such as targeting TGF-β1, PDGF and hyaluronan (HA) synthesis are suggested and some medications are under clinical trials^43,48,49^. Hence, as of now, diagnosis and management of liver fibrosis are still remained a challenge. In current study, transcriptional regulators including miR-34a-5p and CCAT2 were identified as the important controller of TGF-β signaling. It’s also potential biomarker for clinical diagnosis and management of liver fibrosis. However, no directly evidence support that miR-34a-5p and CCAT2 level in liver tissue are correlated with the progress of liver fibrosis.

The fibrogenesis is characterized by pathological ECM accumulation. Remodeling ECM is essential for wound healing in human body, but continuous liver damage result in imbalanced excessive ECM deposition^50^. HSCs are quiescent in normal liver condition. However, when it is activated, establishes new receptors and new proteins expression including platelet derived growth factor (PDGF) receptor, TGF-β receptor, interstitial collagens and α-smooth muscle actin (α-SMA)^51^. As, TGF-β1 is the most critical signaling pathway which regulate fibrosis, inhibiting TGF-β1/Smad pathway is the most relevant way to hold back fibrogenesis progression. Smad2 and Smad3 are R-Smads, and Smad4 is belongs to Co-Smad. In fibrosis activity, binding of phosphorylated Smad2/3 and Smad4 protein is transported into the nucleus, and activates TGF-β1 signaling^53^. Smad4 is also responsible for fibronectin gene expression regulation, so Smad4 is essential for TGF-β1/Smad signaling pathway^52^.

TGF-β1 signaling can be regulated by either lncRNAs^54–56^ or miRNAs^57–59^. Upregulated lncRNA CCAT2 enhances tumor cell growth by controlling TGF-β1 signaling^26^. However, contribution of CCAT2 on fibrosis as a regulator has not been characterized. Administration of inhibited CCAT2 was not only inhibiting HSCs proliferation but also enhancing apoptosis by cell cycle arrestment in the G1 stage. In addition, CCAT2 silencing decreased the FSP1, α-SMA, collagen I and III, which means deregulated ECM accumulation. Moreover, decreased CCAT2 diminished liver fibrosis through preventing phosphorylated Smad2/3, Smad4 and TGF-β1 signaling. To sum up, CCAT2 inhibition could be an effective treatment for liver fibrosis. Similar with the key role of TGF-β signaling pathway in ECM, CCAT2 and miR-34a-5p logically involved in ECM accumulation during fibrogenesis. It suggested that these factor could be potential drug target of liver fibrosis.

In addition, miR-34a-5p was directly binding to 5′-UTR CCAT2 site or to 3′-UTR Smad4 site, and showed significantly decreased dual-luciferase activity. Concerning that binding with miR-34a-5p eliminated their effect, and negatively related with both of them. Thus, CCAT2 is a sponge of miR-34a-5p and reduce its function on Smad4.

In conclusion, CCAT2 play as ceRNA of miR-34a-5p to regulate HSC proliferation via interfering TGF-β1/Smad4 signaling pathway.

## Supplementary Information

Supplementary Information.

## Data Availability

The data that support the findings of this study are available from the corresponding authors on reasonable request.
