# Supplementary material for: LncRNA CCAT2, involving miR-34a/TGF-β1/Smad4 signaling, regulate hepatic stellate cells proliferation
Source: Sci Rep. 2022 Dec 8;12:21199. doi: 10.1038/s41598-022-25738-6 (PMC9732356; doi:10.1038/s41598-022-25738-6)
Supplement: Supplementary file 1 — Supplementary Information. [file 41598_2022_25738_MOESM1_ESM.pdf]

Supplementary Materials for

miR-34a, a physical exercise regulator, involved in LncRNA CCAT2 signaling to r egulate hepatic stellate cells proliferation

Haibing Gao[1,2,*,#], Xiangmei Wang[1,*], Huaxi Ma[1,*], Shenglong Lin[1], Dongqing Zhang[1], WenjunWu [1], Ziyuan Liao[1], Mengyun Chen[1], Hanhui Ye[1], Qin Li[1], Minghua Lin[1,#], Dongliang Li[2,3#]

[1] Mengchao Hepatobiliary Hospital of Fujian Medical University, 350025 Fujian Province, Chin a

[2] Fuzhong Clinical Medical College of Fujian Medical University, 362002 Fujian Province, Chi na

3 900th Hospital of Joint Logistics Support Forces of the Chinese PLA, 350000, Fujian Provin ce, China

[#] Correspond to Dongliang Li (dongliangli93@163.com), Minghua Lin (drlmh543@126.com) and Haibing Gao (drgaohb605@163.com).

*These authors contributed equally to this work.

Supplemental Figure 1. CCAT2 regulates the expression of cycle and apoptosis marker proteins in lx-2 cells. β-actin was used as an internal control. Data are reported as means±SD.*, P<0.05, ** , P<0.01,***, P<0.005.

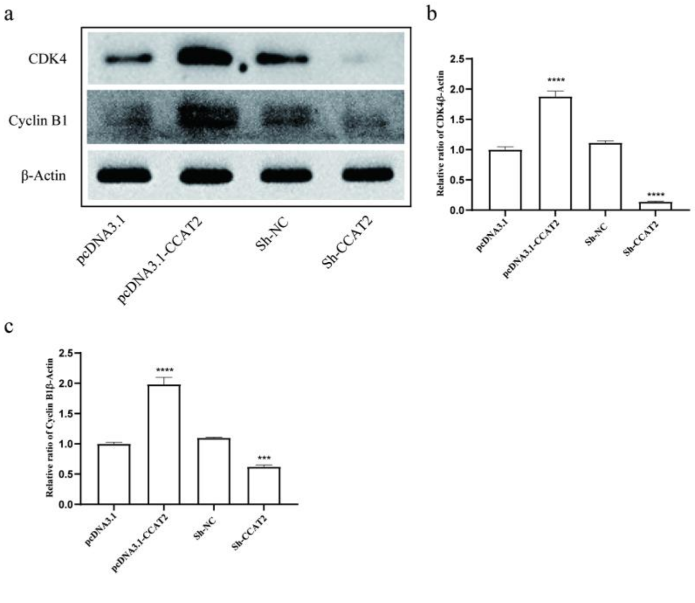

Supplemental Figure 2. miR-34a-5p regulates the expression of cycle and apoptosis marker proteins in HSCs cells. β-actin was used as an internal control. Data are reported as means ±SD.*, P<0.05, ** , P<0.01,***, P<0.005.

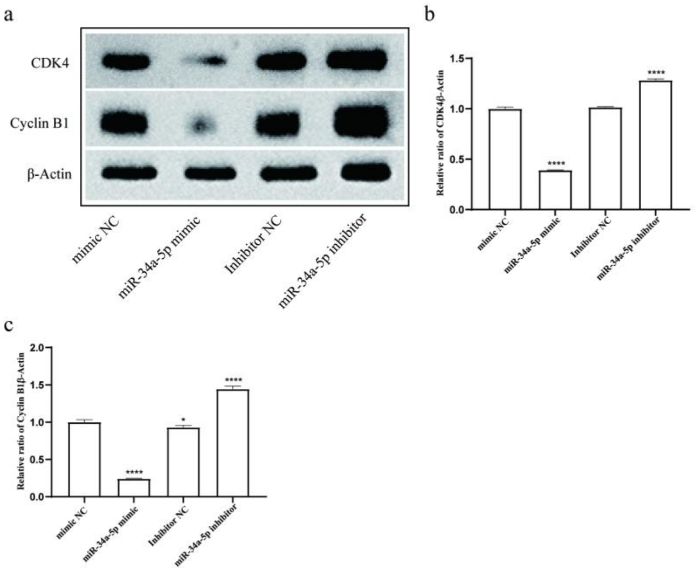

Supplemental Figure 3. CCAT2 control miR34a-5p regulates the expression of cycle and apoptosis marker proteins in HSCs cells. β-actin was used as an internal control. Data are reported as means ± SD.*, P<0.05, ** , P<0.01,***, P<0.005.

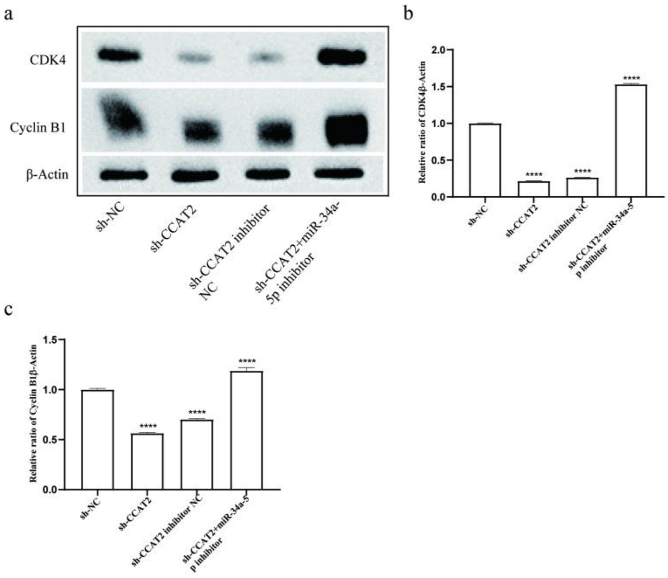

Supplemental Figure 4. TGF-β1 and Smad4 regulates the expression of cycle and apoptosis marker proteins in HSCs cells. β-actin was used as an internal control. Data are reported as means ± SD.*, P<0.05, ** , P<0.01,***, P<0.005.

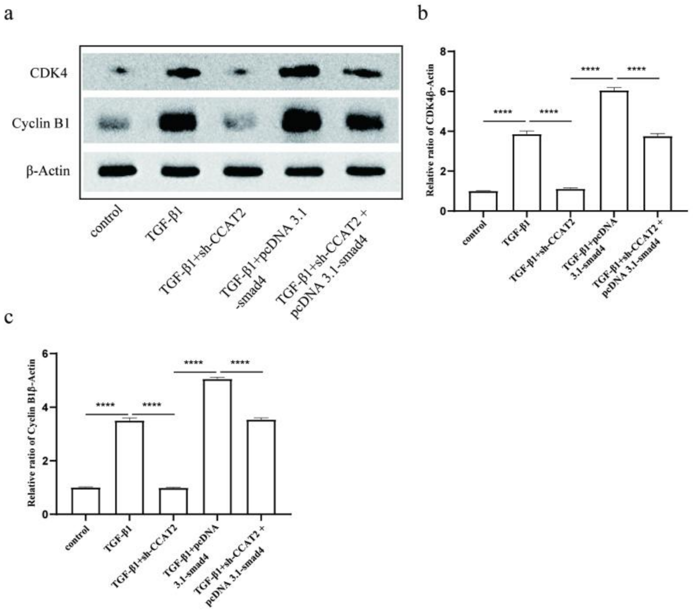

Supplemental Figure 5. Wild type 3'-UTRs of Smad4 and interaction sites between miR-34a-5p. Dual luciferase assay of HSCs co-transfected with Smad4 wild type or mutants and miR-34a-5p mimics or negative control.

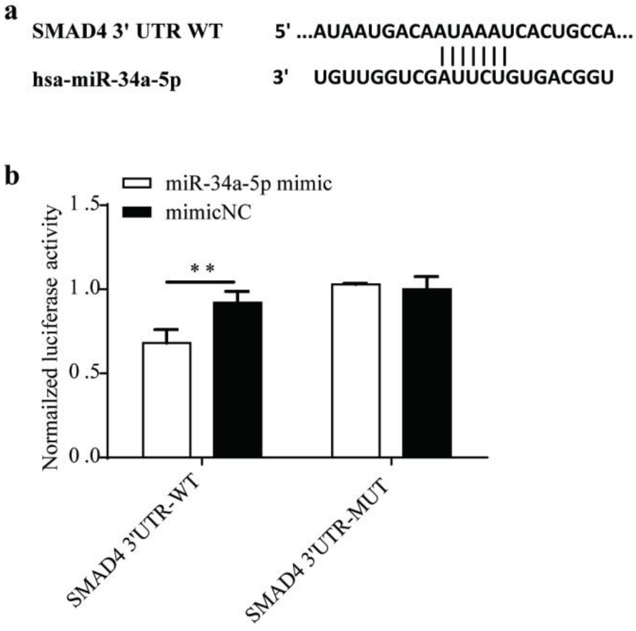

WB original figure

Figure 2a ： β-actin

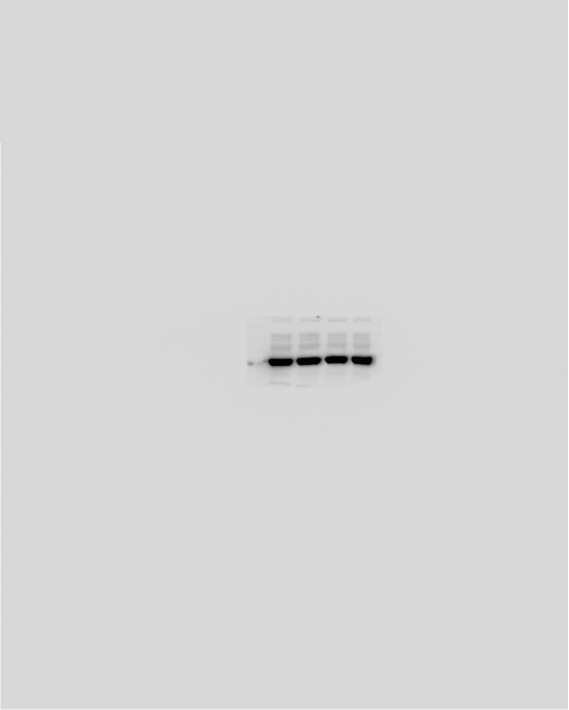

Figure 2a ： Smad4

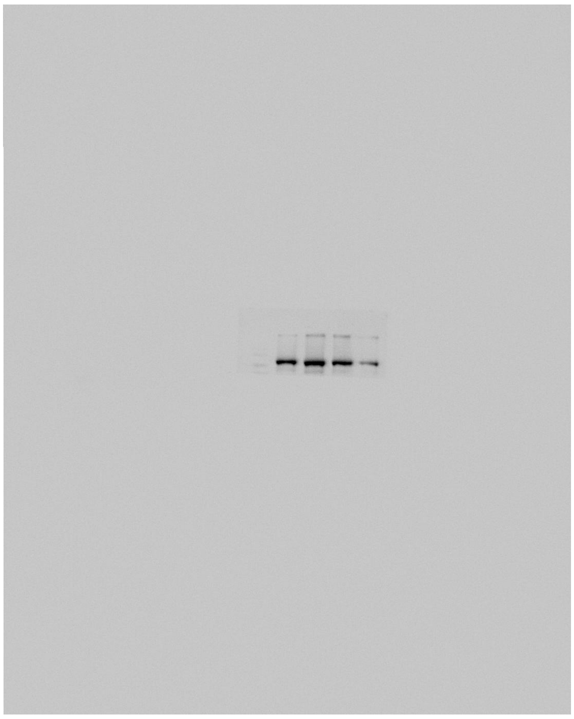

Figure 2b ： β-actin

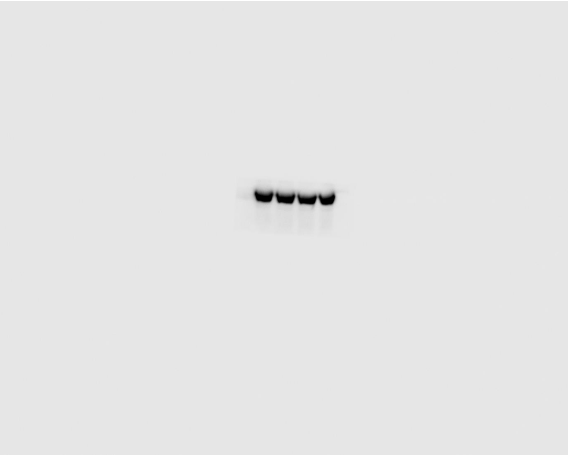

Figure 2b ： Collagen Ⅰ

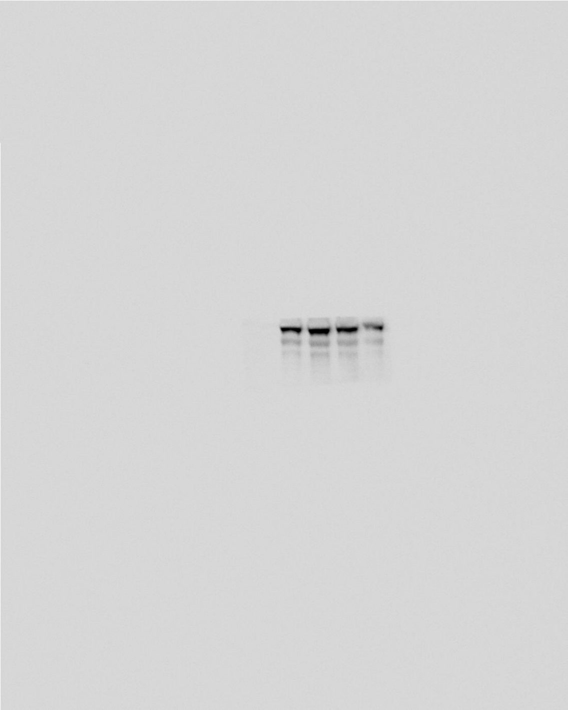

Figure 2b ： Collagen Ⅲ

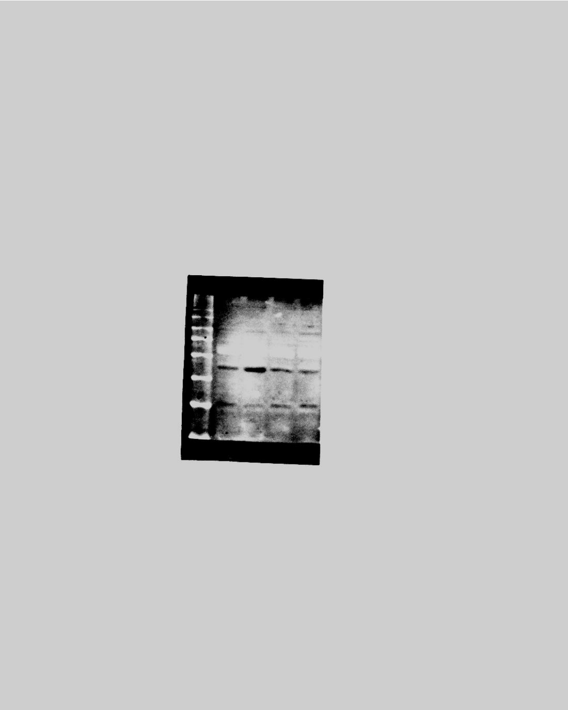

Figure 2b ： FSP1

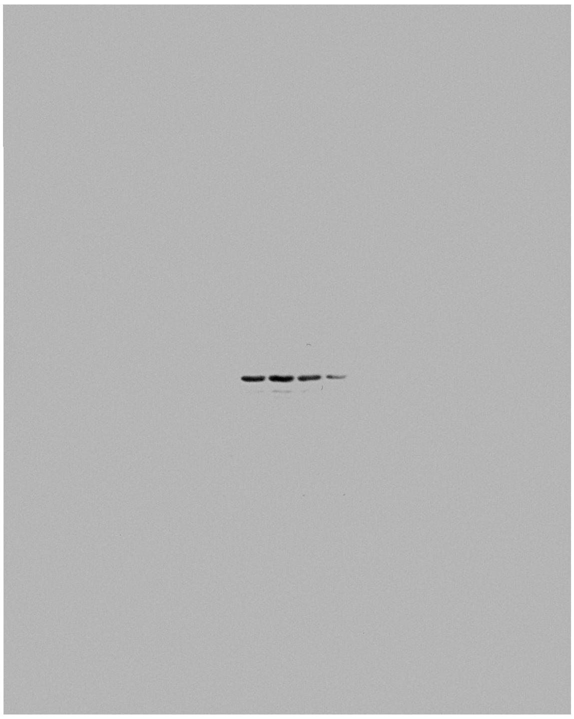

Figure 2b ： P-smad2

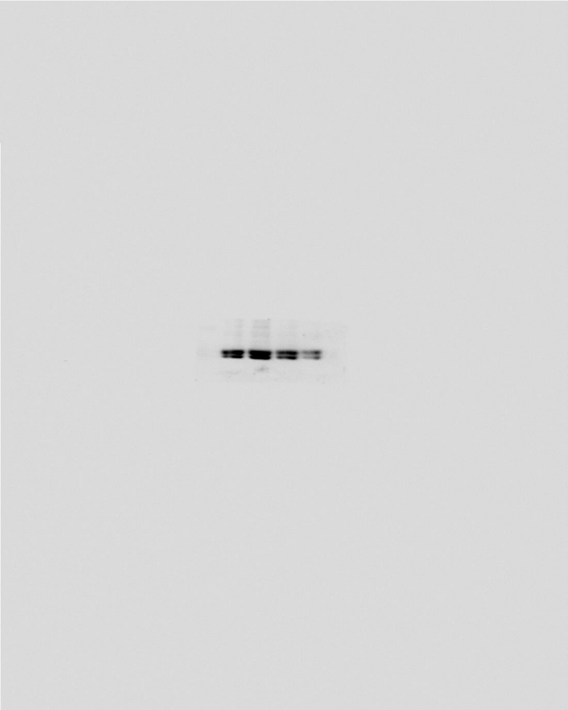

Figure 2b ： smad23

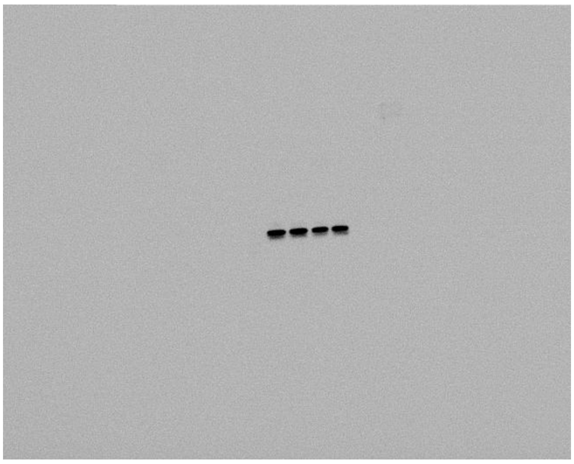

Figure 2b ： α-SMA

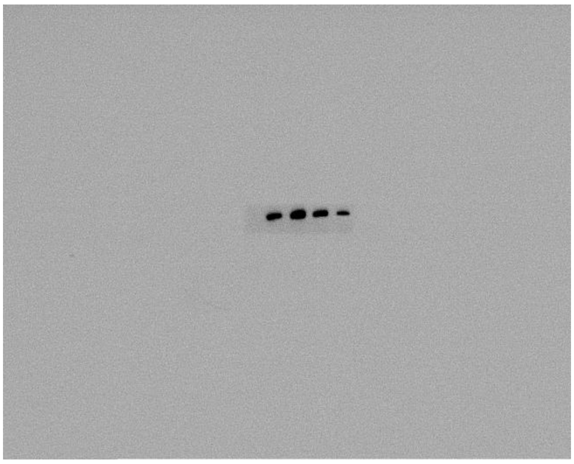

Figure 5a ： β-actin

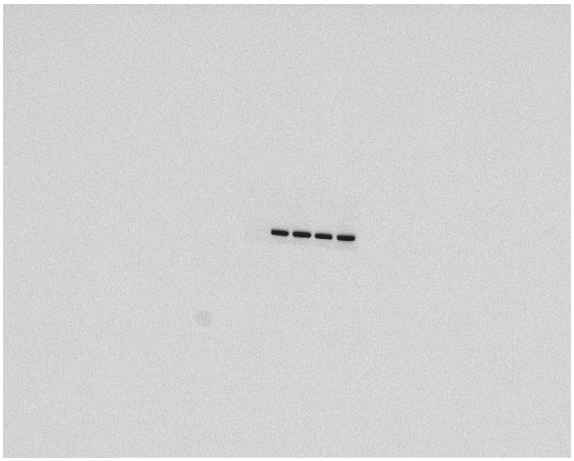

Figure 5a ： smad4

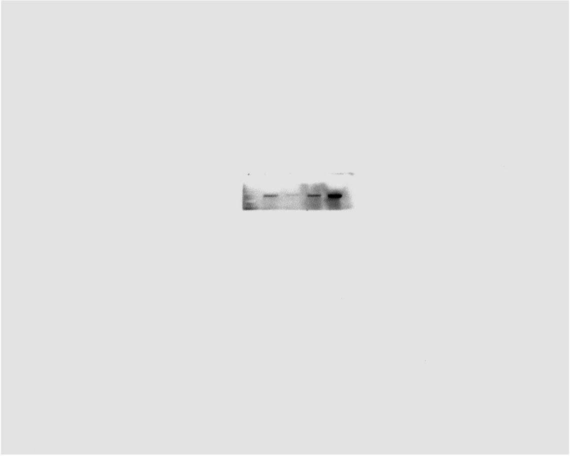

Figure 5b ： β-actin

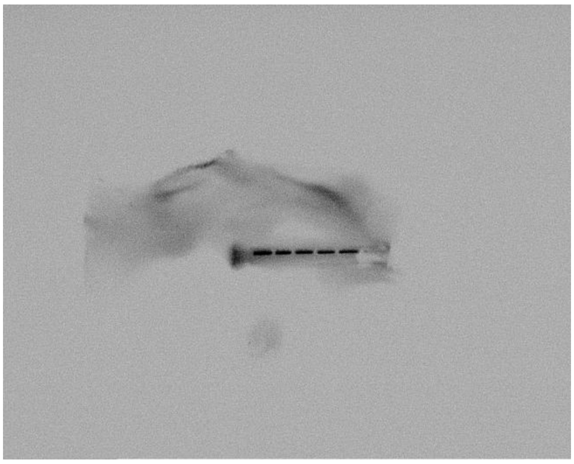

Figure 5b ： Collagen Ⅰ

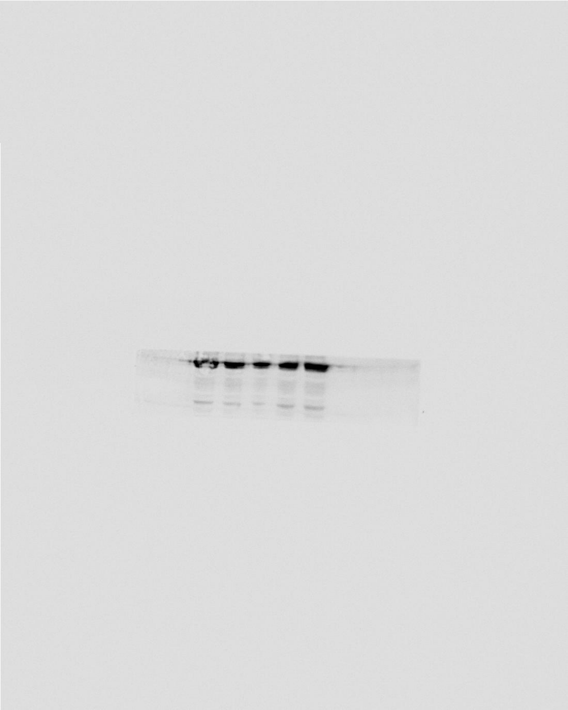

Figure 5b ： Collagen Ⅲ

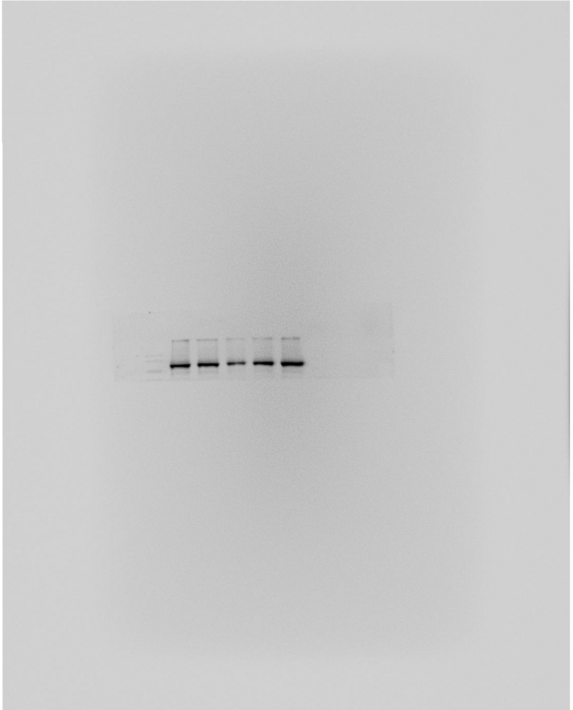

Figure 5b ： FSP1

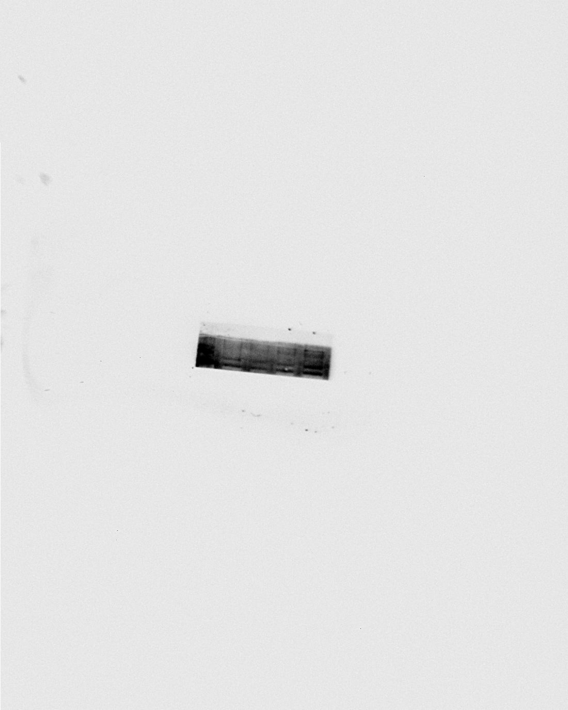

Figure 5b ： P-smad23

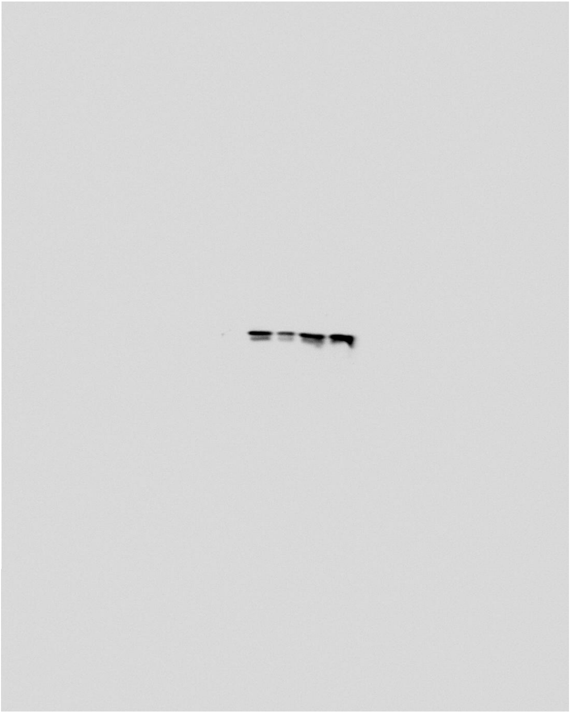

Figure 5b ： smad23

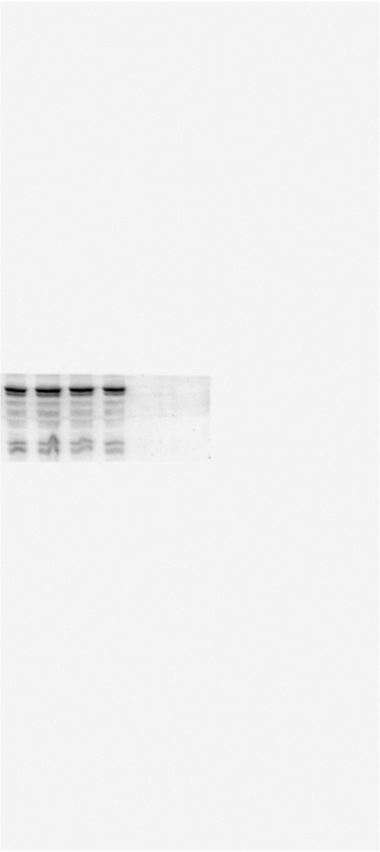

Figure 5b ： α-SMA

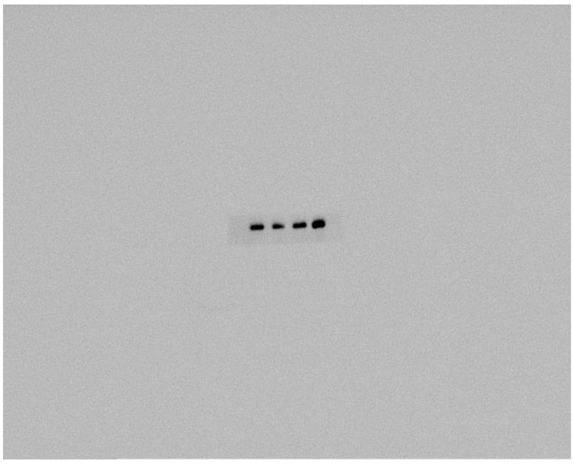

Figure 7a ： β-actin

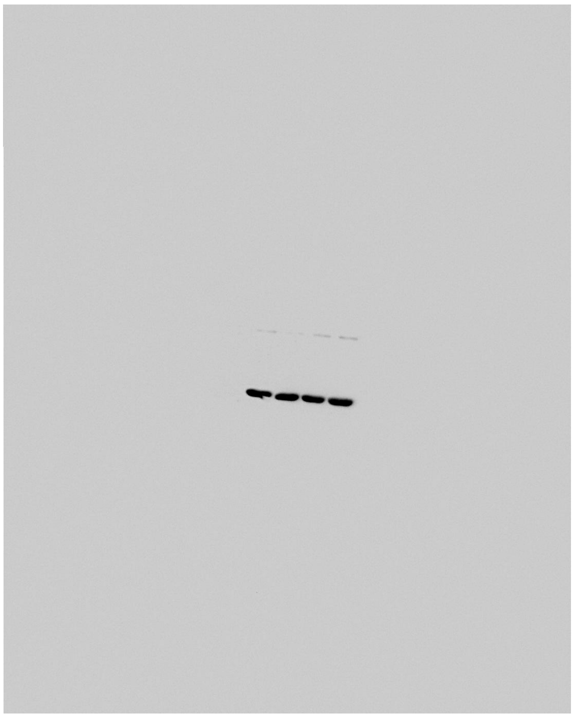

Figure 7a ： smad4

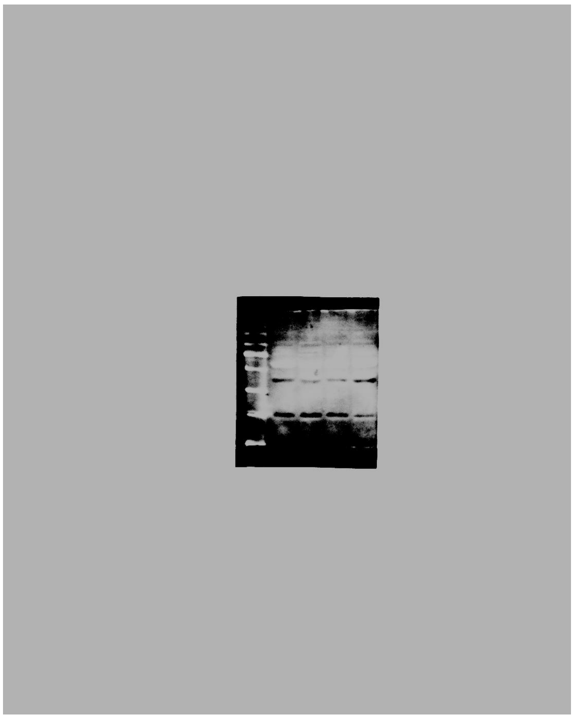

Figure 7b ： β-actin

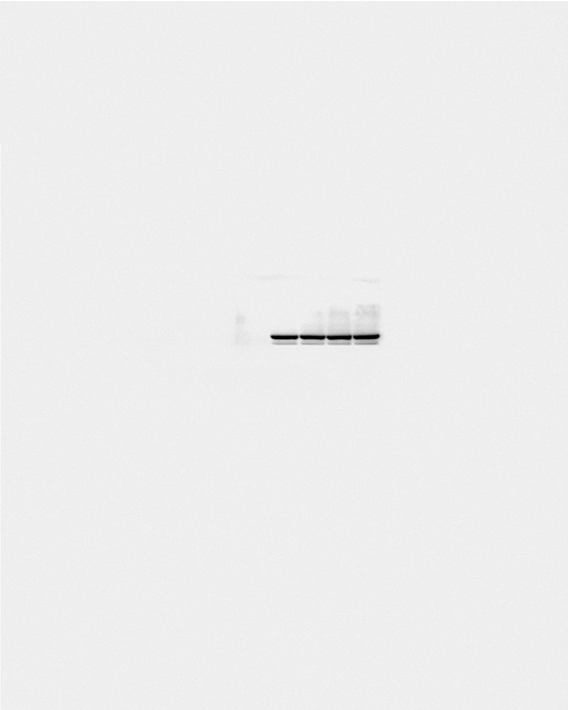

Figure 7b ： Collagen Ⅰ

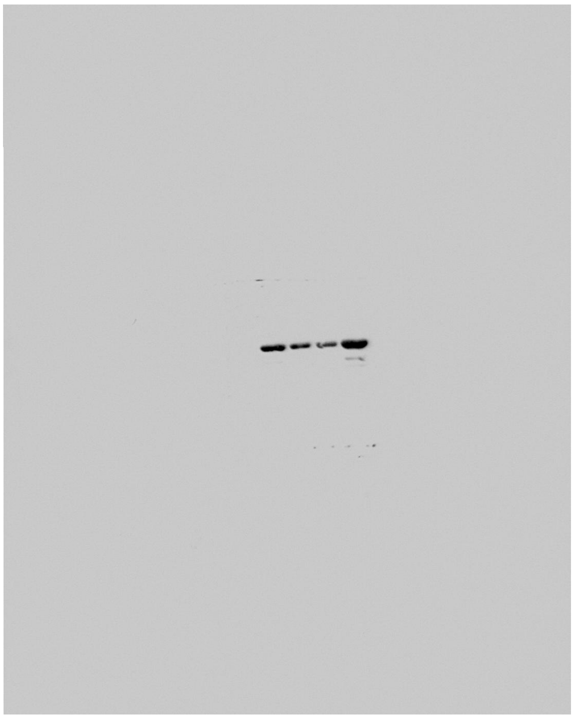

Figure 7b ： Collagen Ⅲ

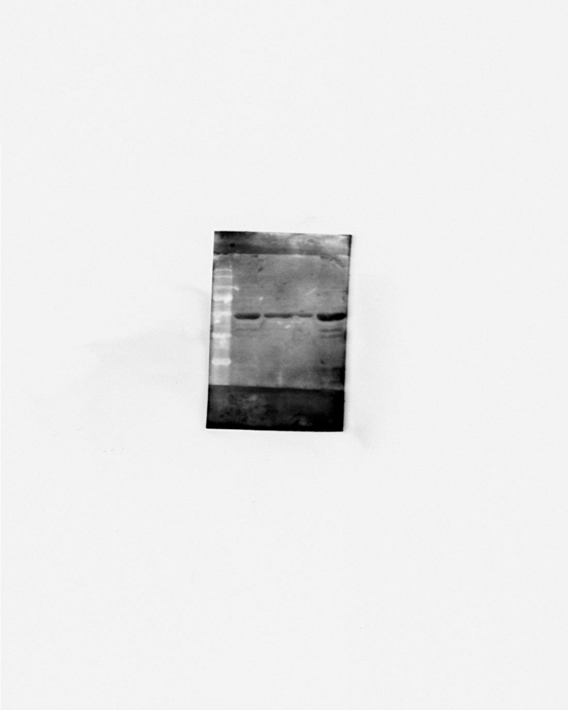

Figure 7b ： FSP1

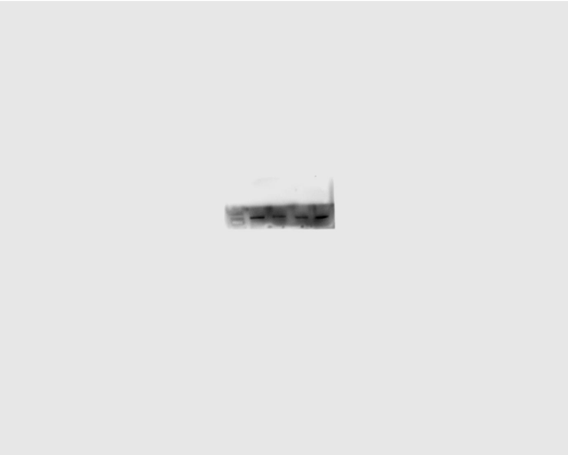

Figure 7b ： P-smad23

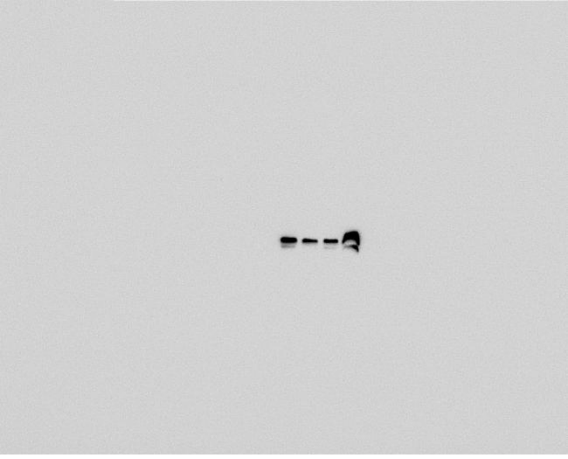

Figure 7b ： smad23

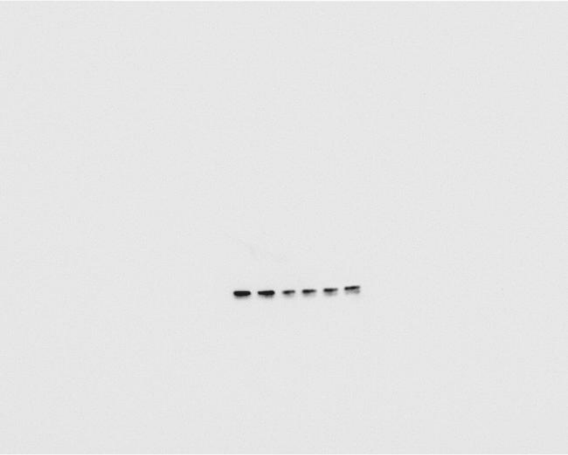

Figure 7b ： α-SMA

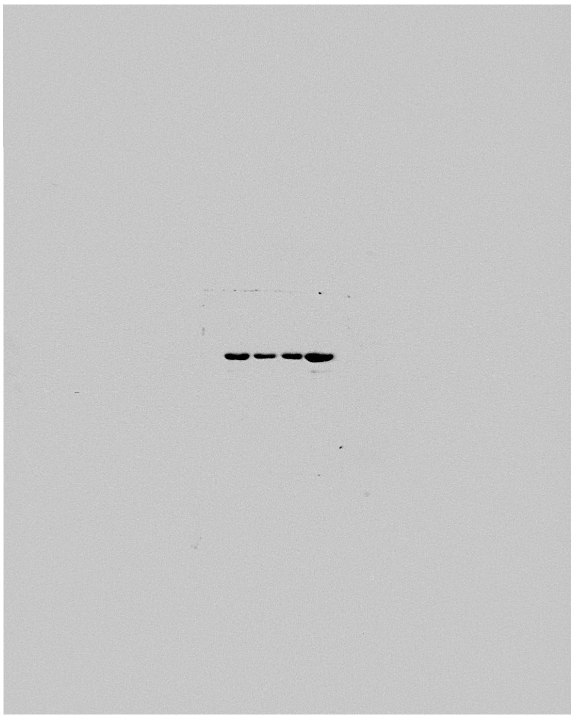

Figure 9a ： β-actin

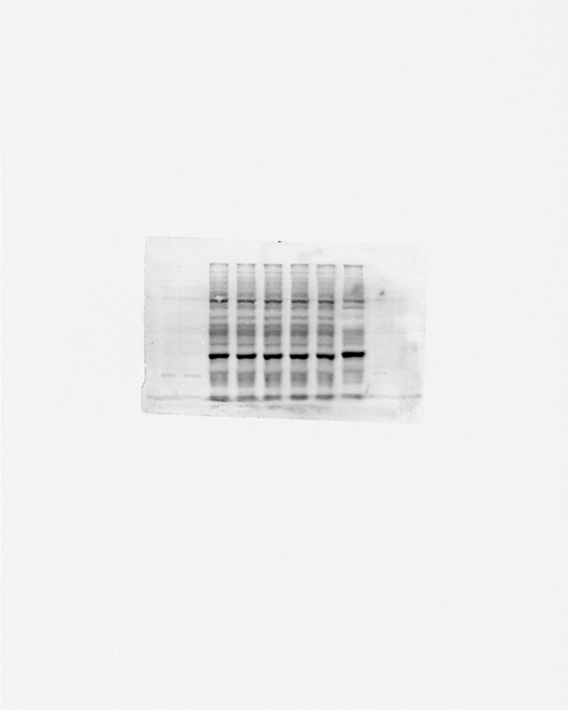

Figure 9a : smad4

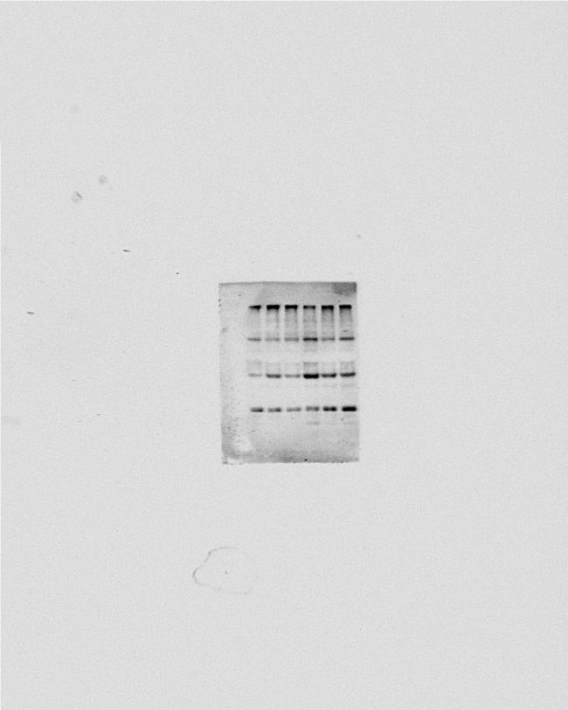

Figure 9b : β-actin

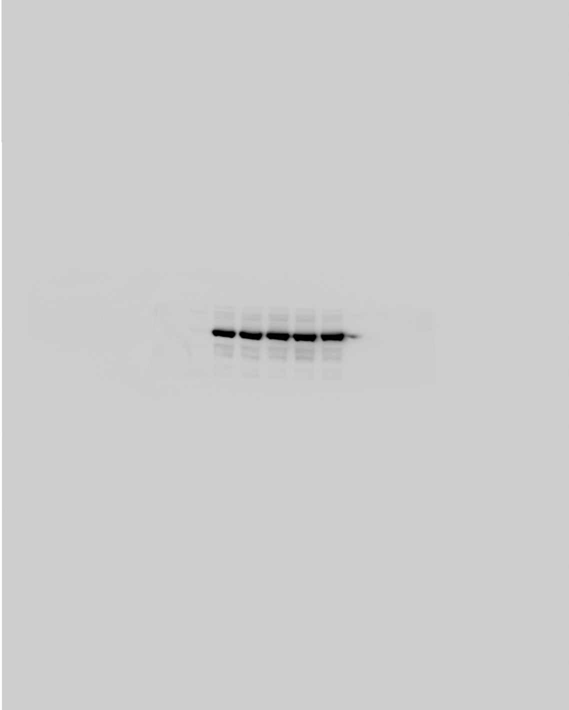

Figure 9b ： Collagen Ⅰ

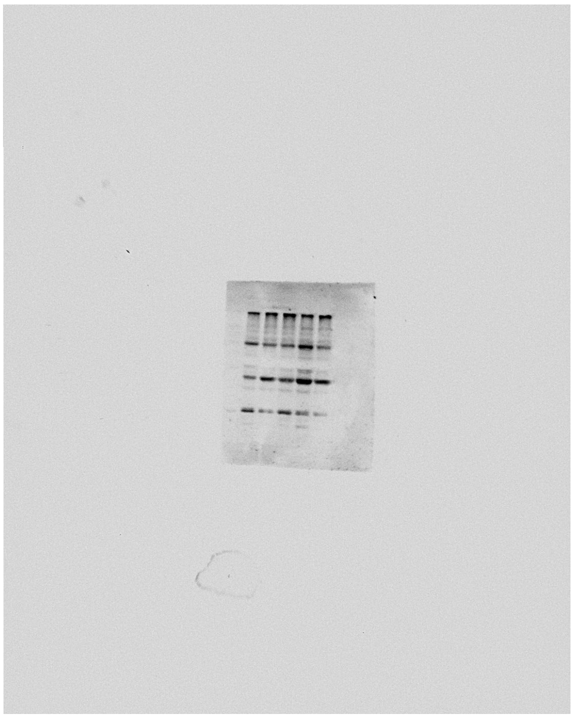

Figure 9b ： Collagen Ⅲ

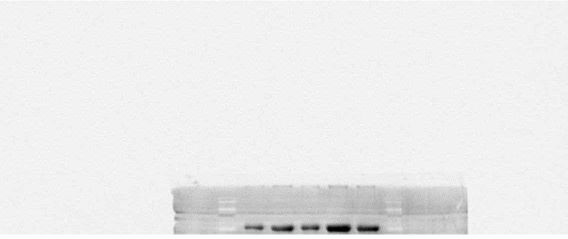

Figure 9b ： FSP1

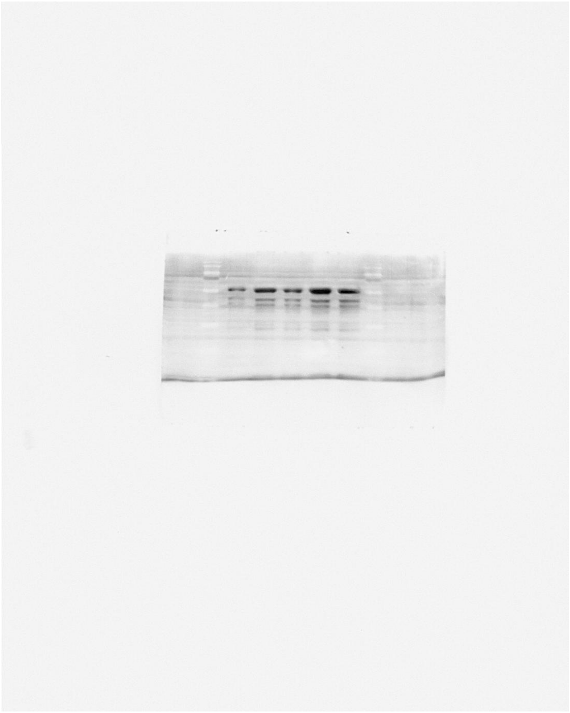

Figure 9b ： P-smad23

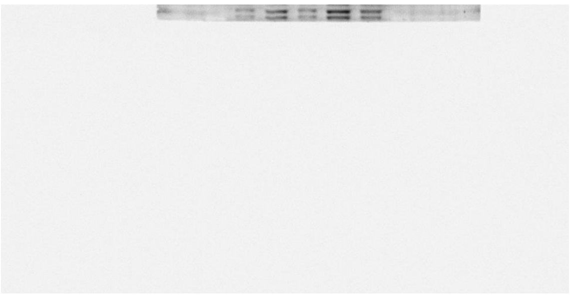

Figure 9b ： smad23

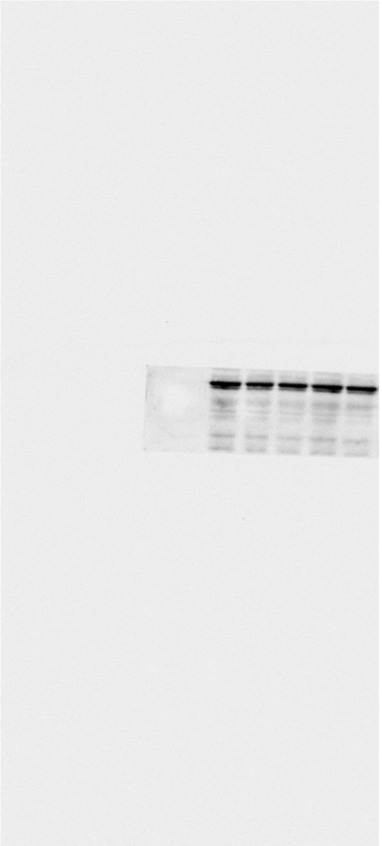

Figure 9b ： α-SMA

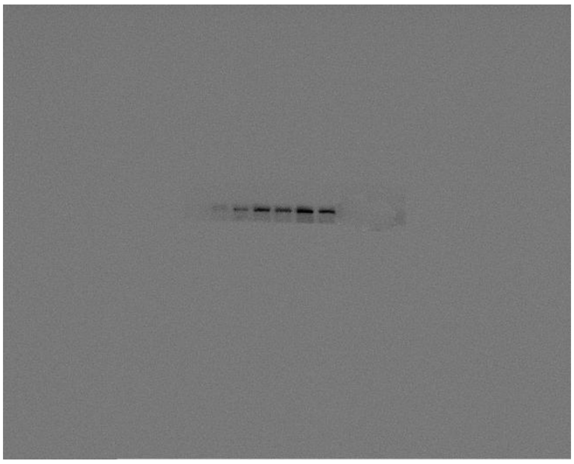

Supplemental Figure 1 ： CDK4

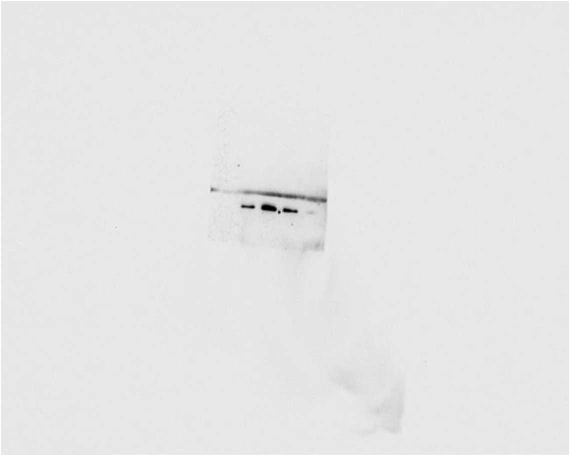

Supplemental Figure 1 ： Cyclin B1

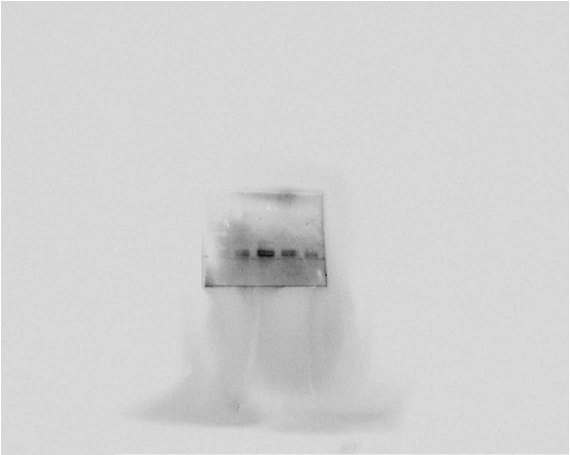

Supplemental Figure 1 ： β-actin

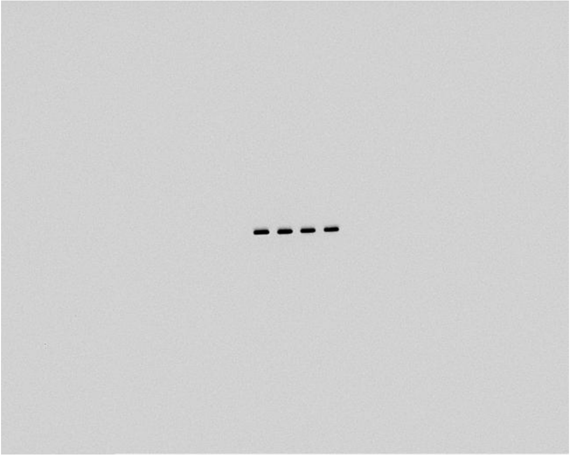

Supplemental Figure 2 ： CDK4

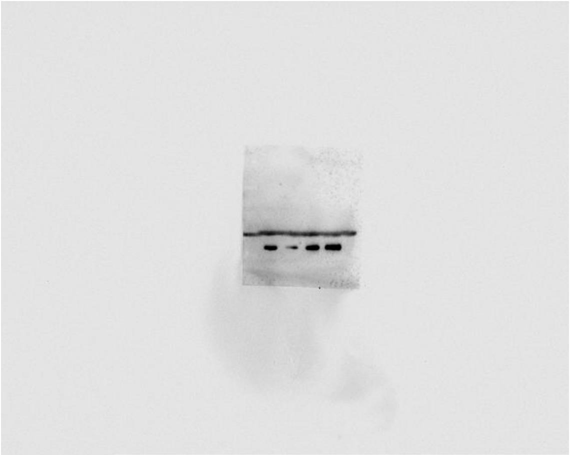

Supplemental Figure 2 ： Cyclin B1

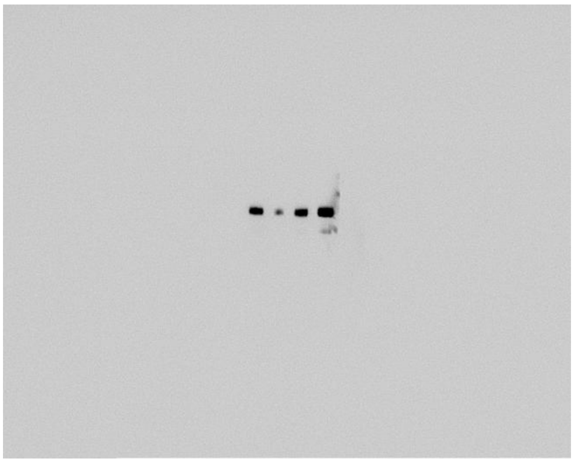

Supplemental Figure 2 ： β-actin

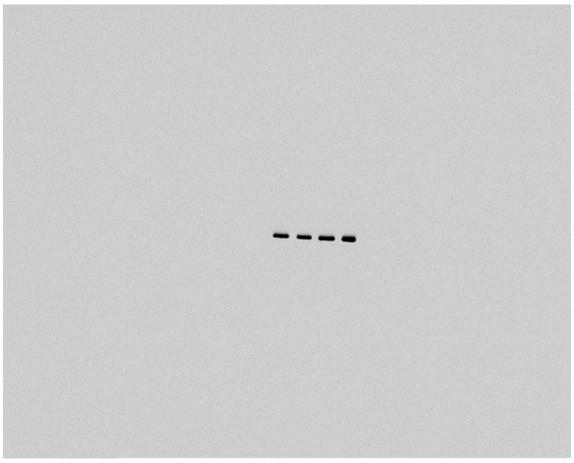

Supplemental Figure 3 ： CDK4

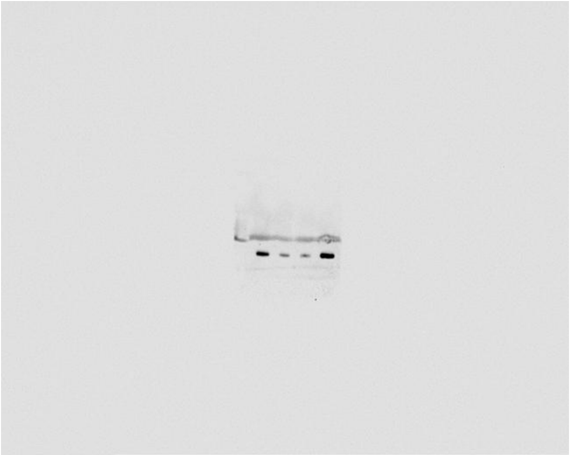

Supplemental Figure 3 ： Cyclin B1

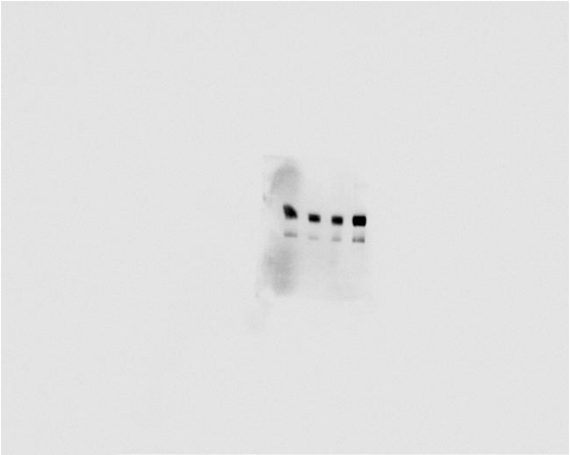

Supplemental Figure 3 ： β-actin

Supplemental Figure 4 ： CDK4

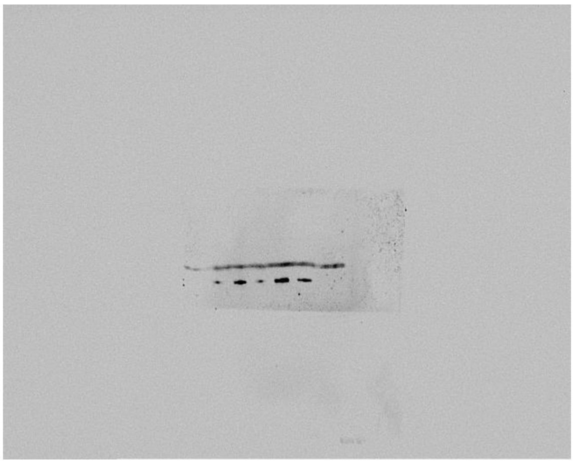

Supplemental Figure 4 ： Cyclin B1

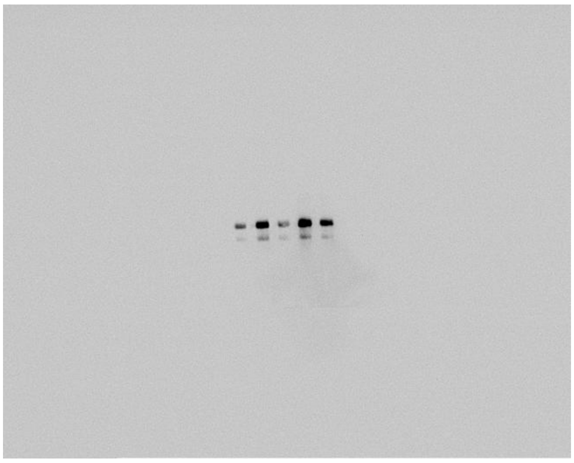

Supplemental Figure 4 ： β-actin
